# Lipid profiling of suction blister fluid: comparison of lipids in interstitial fluid and plasma

**DOI:** 10.1186/s12944-019-1107-3

**Published:** 2019-08-24

**Authors:** Anders K. Nilsson, Ulrika Sjöbom, Karin Christenson, Ann Hellström

**Affiliations:** 1 Section for Ophthalmology, Department of Clinical Neuroscience, Institute of Neuroscience and Physiology, Sahlgrenska Academy, University of Gothenburg, Gothenburg, Sweden; 2 Department of Oral Microbiology and Immunology, The Sahlgrenska Academy, University of Gothenburg, Gothenburg, Sweden; 3 Department of Clinical Neuroscience at Institute of Neuroscience and Physiology, Drottning Silvias Barn- och Ungdomssjukhus, Tillväxtcentrum, Vitaminvägen 21, 416 50 Göteborg, Sweden

**Keywords:** Diacylglycerol, Highly unsaturated fatty acids, Lipidomics, Liquid chromatography–mass spectrometry, Long-chain polyunsaturated fatty acids, Lysophospholipid, Omega-3 fatty acids, Phospholipids

## Abstract

**Background:**

Recent technical advances in the extraction of dermal interstitial fluid (ISF) have stimulated interest in using this rather unexploited biofluid as an alternative to blood for detection and prediction of disease. However, knowledge about the presence of useful biomarkers for health monitoring in ISF is still limited. In this study, we characterized the lipidome of human suction blister fluid (SBF) as a surrogate for pure ISF and compared it to that of plasma.

**Methods:**

Plasma and SBF samples were obtained from 18 healthy human volunteers after an overnight fast. Total lipids were extracted and analyzed by liquid chromatography-tandem mass spectrometry. One hundred ninety-three lipid species covering 10 complex lipid classes were detected and quantified in both plasma and SBF using multiple reaction monitoring. A fraction of the lipid extract was subjected to alkaline transesterification and fatty acid methyl esters were analyzed by gas chromatography–mass spectrometry.

**Results:**

The total concentration of lipids in SBF was 17% of the plasma lipid concentration. The molar fraction of lipid species within lipid classes, as well as total fatty acids, showed a generally high correlation between plasma and SBF. However, SBF had larger fractions of lysophospholipids and diglycerides relative to plasma, and consequently less diacylphospholipids and triglycerides. Principal component analysis revealed that the interindividual variation in SBF lipid profiles was considerably larger than the within-subject variation between plasma and SBF.

**Conclusions:**

Plasma and SBF lipid profiles show high correlation and SBF could be used interchangeably with blood for the analysis of major lipids used in health monitoring.

**Electronic supplementary material:**

The online version of this article (10.1186/s12944-019-1107-3) contains supplementary material, which is available to authorized users.

## Introduction

The interstitial space in tissue is filled with interstitial fluid (ISF), which enables cell-to-cell interactions and provides a matrix for the transport of nutrients and metabolic waste between cells and capillaries. The biomolecular composition of ISF is consequently influenced by the characteristics of the tissue as well as the plasma. Although ISF constitutes up to 25% of humans’ total body weight [1], extraction of ISF for biomarker analysis has proved challenging. However, recent technical advances allowed the development of a new generation of minimally invasive devices aimed at extracting ISF, and thereby evoked a resurgence of interest in this matrix for health status monitoring. Approaches using microneedles to extract dermal ISF seem particularly promising [2]. Microneedles have been demonstrated to extract an analytical volume of dermal ISF from humans that is sufficient for determining transcriptomic [3] and proteomic signatures [3, 4] as well as other biomarkers [5]. Several advantages of using ISF over blood (i.e., plasma, serum or whole blood) for biomarker analysis have been proposed. ISF is a relatively simple matrix compared to blood, allowing more facile isolation and characterization of certain molecular compounds. Further, because the composition of ISF reflects that of surrounding cells, the status of the tissue on a local scale could be retrieved without the need for biopsies [6]. Certain patient groups in particular may benefit from replacing sampling of blood with ISF. For example, phlebotomy blood loss for clinical monitoring of very and extremely low-birth-weight infants is associated with anemia and morbidities [7–10]. Thus, replacing some blood samples with ISF samples in ill neonates could alleviate blood loss, limit the number of blood transfusions, and potentially reduce the frequency and/or severity of morbidities.

Suction blister fluid (SBF) largely consists of ISF and has been used as a substitute for true ISF. When a mild negative pressure is applied to a small skin area for 1 to 3 h, the epidermis and dermis separate and fluid-filled blisters are formed [11]. The fluid contained inside the blister can then be collected using a needle and syringe. This simple technique yields rather large fluid volumes (typically 100–200 μl) compared to the current microneedle system, and may therefore be used to determine basic characterization of ISF and its potential use as an alternative to blood. The molecular composition of SBF has been studied using various methods, including proteomics [12–14] and metabolomics [15, 16]. These studies show a high overlap in composition between plasma/serum and SBF, indicating that ISF could function as a proxy for blood sampling.

The concentration of lipids in SBF on a lipid class level were determined 40 years ago [17]. Since then, very little attention has been paid to the lipidome of SBF. The composition of lipids in SBF at a molecular species level and how it relates to that of blood/plasma have not been investigated.

Lipids play essential roles in numerous cellular processes, and their potential as a source of biomarkers for disease detection and prediction is being increasingly recognized [18]. Lipids are transported in the blood in the form of lipoproteins and enter the ISF by diffusion through the vascular wall [19]. Several diseases and metabolic syndromes, including cancer, autoimmunity, cardiovascular disease, obesity, and diabetes, have been connected to alterations in blood lipid profiles. A group of lipids that have received special attention over the last decades are the omega-3 (n-3) long-chain polyunsaturated fatty acids (LC-PUFA), particularly regarding how these relate proportionally to omega-6 LC-PUFAs in blood and tissue [20]. A high fraction of n-3 LC-PUFAs have been suggested to positively contribute to cognition [21] and prevent disease [20, 22, 23].

The present report concerns the distribution of lipids and fatty acids in SBF in relation to plasma. We show an overall high resemblance of the SBF lipidome to plasma and propose that ISF could be used interchangeably with blood to determine the status of LC-PUFA and other lipids.

## Methods

### Sample collection

Plasma and SBF samples were obtained from 18 healthy human volunteers (13 females and 5 males, mean age 50.3 years; min/max 29.0/64.3 years) after an overnight fast. For plasma samples, whole blood was obtained by venipuncture and collected into 4-mL K_2_EDTA tubes (VACUETTE®, Greiner Bio-One GmbH, Kremsmünster, Austria). The tubes were kept at room temperature for 15 min before centrifugation at 4 °C and 2000 *g* for 15 min. Plasma aliquots were transferred to 0.5-mL cryotubes and stored at − 80 °C until analysis. SBF was collected from suction blisters generated on the forearm as previously described [24].

### Lipid extraction

Total lipid extract was obtained from plasma/SBF using a modified protocol of the BUME method [25]. Briefly, 25 μL plasma or SBF was spiked with SPLASH® LIPIDOMIX® Mass Spec Standard (Avanti Polar Lipids, Alabaster, AL, U.S.) containing PC 15:0–18:1(d7), PE 15:0–18:1(d7), PS 15:0–18:1(d7), PI 15:0–18:1(d7), PC-O 18(Plasm)-18:1(d9), PE-O C18(Plasm)-18:1(d9), LPC 18:1(d7), LPE 18:1(d7), CE 18:1(d7), DG 15:0–18:1(d7), TG 15:0–18:1(d7)-15:0, SM 18:1(d9) and extracted in ice cold butanol:methanol (3:1) with butylated hydroxy toluene (BHT; 0.01% w/v) for 30 min in an ultrasonicator. Phase separation was achieved by additing heptane:ethylacetate (3:1) containing 1% acetic acid. After 30 s vortexing and 10 min centrifugation at 1000 *g*, the top organic layer was transferred to a new glass tube. The original tube was re-extracted twice with heptane:ethylacetate 1% acetic acid according to this procedure. The pooled extracts were evaporated to dryness under a stream of N_2_. Samples were dissolved in chloroform:methanol (2:1) and split into two equal volumes. Finally, the solvent was evaporated and the sample reconstituted in 100/25 μL (plasma/SBF) isopropanol for analysis of CEs and methanol for analysis of all other lipid classes.

### Liquid chromatography mass spectrometry

Lipid samples were analyzed on an Agilent 1260 Infinity HPLC system coupled to an Agilent 6410 triple quadrupole mass spectrometer (Agilent Technologies, Santa Clara, CA) equipped with an electrospray ionization source. Chromatographic separation was achieved on an Acclaim C30 2.1 × 50 mm 3-μm analytical column connected to a 2.1 × 10 mm 5-μm guard cartridge (both ThermoFisher Scientific, Thermo Electron Sweden AB, Hägersten, Sweden) thermostated to 50 °C. Mobile phase A consisted of acetonitrile:methanol:water (35:35:30 by volume) and B isopropanol; both were supplemented with 0.1% formic acid and 0.05% ammonia for analyses in positive mode and with 10 mM ammonium acetate for analyses in negative mode. Mobile phases were eluted at a constant flow of 0.35 mL·min^− 1^ starting with isocratic elution of 100% A for 5 min, a linear increase to 95% B in 25 min, isocratic elution for 5 min followed by reversal to 100% A, and finally column re-equilibration for 8 min. For analysis of CEs, a shorter gradient was used: isocratic elution of 100% A for 4 min, a linear increase to 95% B in 12 min, isocratic elution for 6 min, followed by reversal to 100% A and re-equilibration for 8 min. All solvents were of HPLC grade or higher. The electrospray ion source was operated at a temperature of 250 °C using nitrogen gas at a flow of 4 L·min^− 1^ and nebulizer pressure 30 psi. The capillary voltage was set to 4000(+)/4500(−) V. Scan data was collected with mass range *m*/*z* 400–1000 in positive and *m*/*z* 100–1000 in negative mode. Selected lipids were detected with multiple reaction monitoring (MRM) in positive mode; precursor and product ions are reported in Additional file 4: Table S1. Fragmentor and collision energies were determined for each lipid class. Due to the complex fragmentation patterns of DGs and TGs, both MS1 and MS2 were set to select the parent ion. PE plasmalogens were identified using the diagnostic fragments *m*/*z* 364, 392, and 390 containing 16:0p, 18:0p, and 18:1p, respectively [26]. Peaks were integrated in MassHunter Workstation Software Quantitative Analysis for QQQ (version B.06.00, Agilent Technologies, USA) and quantified against the internal standard of the respective lipid class. Because we were unable to satisfactorily integrate the IS for PEp in many samples, the PE IS was used as a surrogate for this lipid class. Six-point response curves were made for PC 16:0/16:0, PC 18:2/18:2, PC 16:0/20:4, PC 16:0/22:6, and CE 18:2 (Avanti Polar Lipids, Alabaster, AL, U.S., and Larodan AB, Solna, Sweden) to confirm that they were within linear range (weight 1/x, *R*^*2*^ ≥ 0.98). A QC sample from pooled plasma was run every seventh study sample. Out of the 193 quantified lipids, the within-run CV was < 5% for 136 lipid species and < 10% for 174 lipid species (*n* = 5).

### Gas chromatography mass spectrometry

After analysis of CEs, the remaining sample was dried under N_2_ and subjected to alkaline transesterification using sodium methoxide [27]. Fatty acid methyl esters (FAMEs) were analyzed by gas chromatography–mass spectrometry using an Agilent 7820 gas chromatograph coupled to an Agilent 5975 mass selective detector (Agilent Technologies, USA) [28]. FAMEs were identified by comparing retention time and mass spectra to authentic standards (FAME mixture ME 100, stearidonic acid, adrenic acid, osbond acid, and clupanodonic acid, all from Larodan AB, Solna, Sweden). Data was normalized to the internal standard oleic acid (d7) and quantified in MassHunter Workstation Software Quantitative Analysis for GCMS (version B.06.00, Agilent Technologies, USA) using single ion monitoring and response curves built from the FAME standards listed above. Curve weighting 1*/*x was applied to all calibration curves with *R*^*2*^ > 0.99. The within-run CVs ranged between 0.7% (stearic acid, 18:0) and 8.9% (clupanodonic acid, 22:5 n-3) (*n* = 8).

### Statistical analyses

Multivariate data analyses were performed using SIMCA 15 (Umetrics AB, Umeå, Sweden) and univariate statistical analyses were performed using IBM SPSS Statistics, version 25 (IBM Corp, Armonk NY, USA). Correlation analyses were performed using RStudio [29] and the Corrplot package [30].

## Results

### Comparison of lipid profiles in plasma and suction blister fluid

**Fig. 1:**
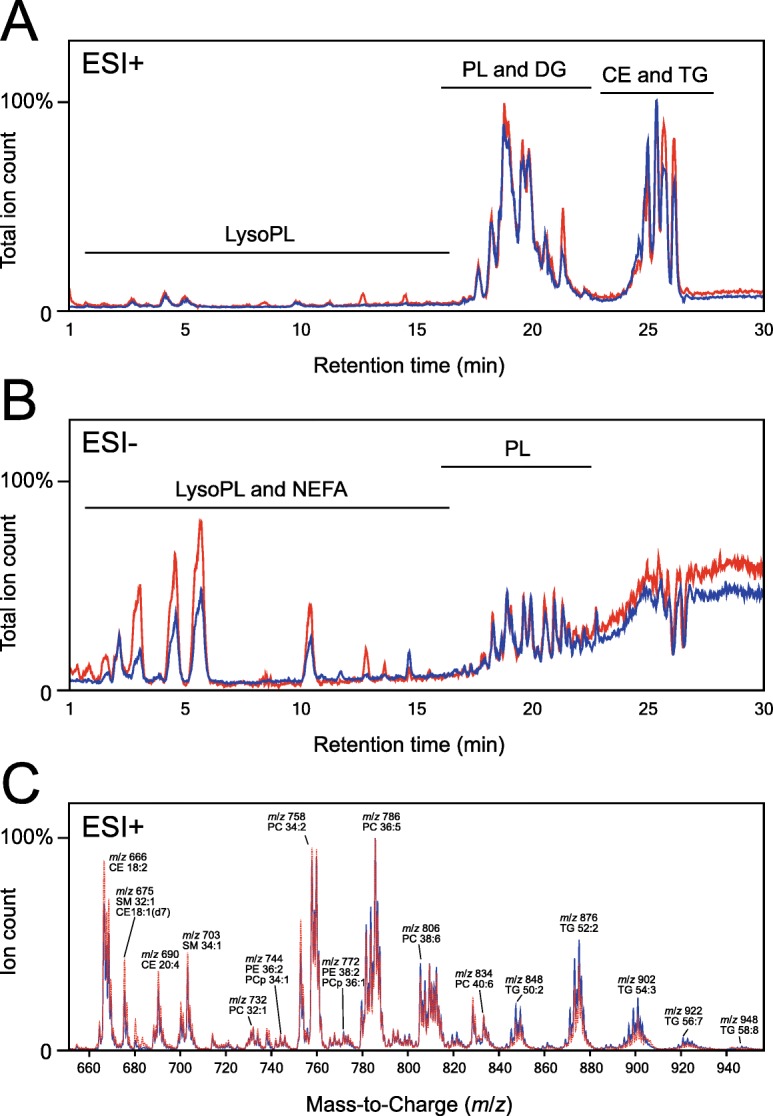
LC-MS/MS scan data of lipid extracts from pooled samples of plasma or suction blister fluid. Total ion chromatograms acquired in positive electrospray ionization (ESI+) (**a**) or negative (ESI-) (**b**) ionization mode. Chromatograms are normalized to the highest peak intensity for ESI+ and to the highest phospholipid peak for ESI-. **c** Mass spectra (range *m*/*z* 650–960) from a total ion chromatogram acquired in positive (ESI+) mode. Major putative lipid species and their corresponding mass-to-charge ratio are indicated above peaks. Blue traces represent plasma and red traces represent SBF in all panels. CE, cholesteryl ester; DG, diglyceride; NEFA, non-esterified fatty acid; LysoPL, lysophospholipid; PL, phospholipid; TG, triglyceride

To compare the overall lipid profiles of plasma and SBF, a total lipid extract was prepared from pooled plasma or SBF samples from 18 healthy individuals and analyzed by liquid chromatography–mass spectrometry (LC-MS) in MS2 scan mode with positive (ESI+)(Fig. 1a) and negative (ESI-) ionization (Fig. 1b). Analysis in positive mode primarily yielded [M + H]^+^ ions for phospholipids and [M + NH_4_]^+^ for neutral lipids, including cholesteryl esters (CEs) and di- and triglycerides (DGs and TGs, respectively). In negative mode, phosphatidylcholines (PCs) and sphingomyelins (SMs) formed [M + OAc]^−^ ions while other phospholipids (including phosphatidylinositol, PI, and phosphatidylserine, PS) and non-esterified fatty acids (NEFAs) were found as [M-H]^−^. The low tendency of CEs and TGs to form negative ions resulted in substantial ion suppression around retention time 25 min. Figure 1c shows a mass spectrum from ESI+ scan data with the major putative lipid species within each mass cluster. Total ion chromatograms, as well as total mass spectra, were similar for the two types of body fluids, and differences appeared to be quantitative rather than qualitative. Based on putative lipid species identified from the scan data, ion transitions for multiple reaction monitoring (MRM) in positive mode were selected. In addition to lipids derived from the scan data, we also included other lipid species known to be present in human plasma in relatively high abundance [31–35].

### Concentration of lipids in plasma and suction blister fluid

**Fig. 2:**
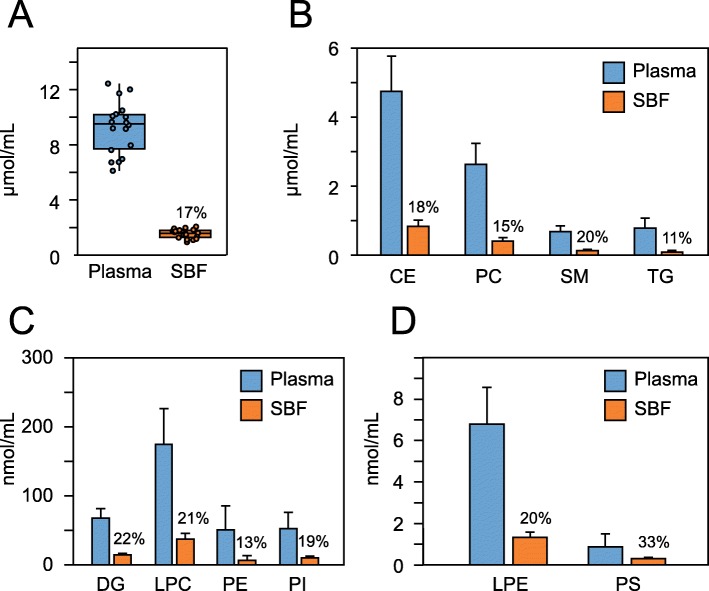
Concentration of lipid classes in plasma and SBF from 18 healthy individuals. **a** Box plot showing the total sum of quantified lipids (in micromoles per milliliter plasma or SBF). The sum of the most concentrated (micromoles per milliliter) (**b**) and least concentrated (nanomoles per milliliter) (**c** and **d**) lipid classes. Numbers above SBF bars show the concentration of lipids in SBF compared to plasma in percent. Mean and SD are shown in B-D (*n* = 18). CE, cholesteryl ester; DG, diglyceride; LPC, lysophosphatidylcholine; LPE, lysophosphatidylethanolamine; PC, phosphatidylcholine; PE, phosphatidylethanolamine; PI, phosphatidylinositol; PS, phosphatidylserine; SM, sphingomyelin; TG, triglyceride

In total, a targeted analysis was performed for 237 lipids in plasma and SBF from 18 individuals (Additional file 4: Table S1). Out of the targeted lipids, 193 lipid species covering 10 complex lipid classes provided good peaks that could be integrated and quantified (Additional file 5: Table S2). The sum of all quantified lipids was 9.2 ± 1.9 (mean ± SD) μmol·mL^− 1^ and 1.5 ± 0.3 μmol·mL^− 1^ for plasma and SBF, respectively (Fig. 2a). The proportion of plasma lipids to SBF lipids was similar in all classes and ranged between 11 and 22%, with the exception of PS which was present at 33% of the plasma level in SBF (Fig. 2b-d). However, PS abundance was (as expected) low, and only two species were included in the analysis.

### Relative distribution of lipid species in plasma and suction blister fluid

**Fig. 3:**
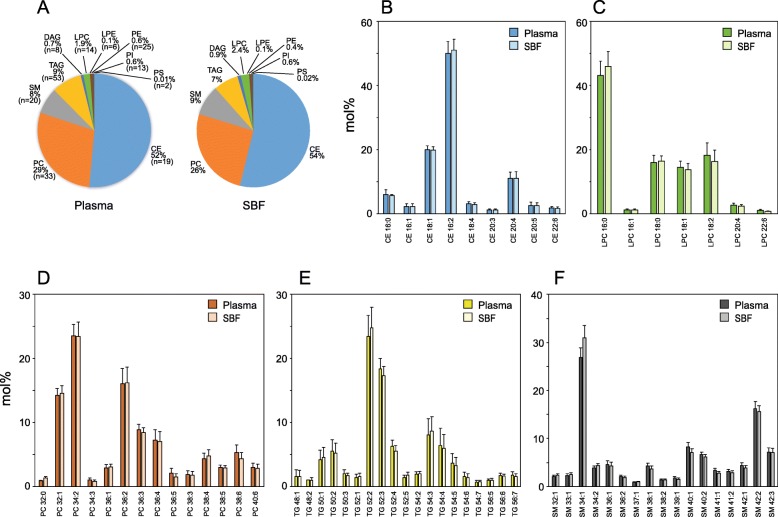
Relative abundance of lipids in plasma and SBF. (**a**) Lipid class composition in plasma and SBF with respect to concentration. The molar percent of lipid species found within lipid classes CE (**b**), LPC (**c**), PC (**d)**, TG (**e**), and SM (**f**). Only lipid species exceeding 1 molar percent are shown. Mean and SD are shown in B-F (*n* = 18). CE, cholesteryl ester; DG, diglyceride; LPC, lysophosphatidylcholine; LPE, lysophosphatidylethanolamine; PC, phosphatidylcholine; PE, phosphatidylethanolamine; PI, phosphatidylinositol; PS, phosphatidylserine; SM, sphingomyelin; TG, triglyceride

There were small but significant differences in the molar distribution of all analyzed lipid classes between plasma and SBF (Student’s *t*-test or Mann-Whitney *U* test, *p* < 0.05, Fig. 3a). The fractions of lipid species within each class were highly similar between plasma and SBF (shown for lipid species above 1 molar percent in Fig. 3b-f and Additional file 1: Figure S1A-D).

**Fig. 4:**
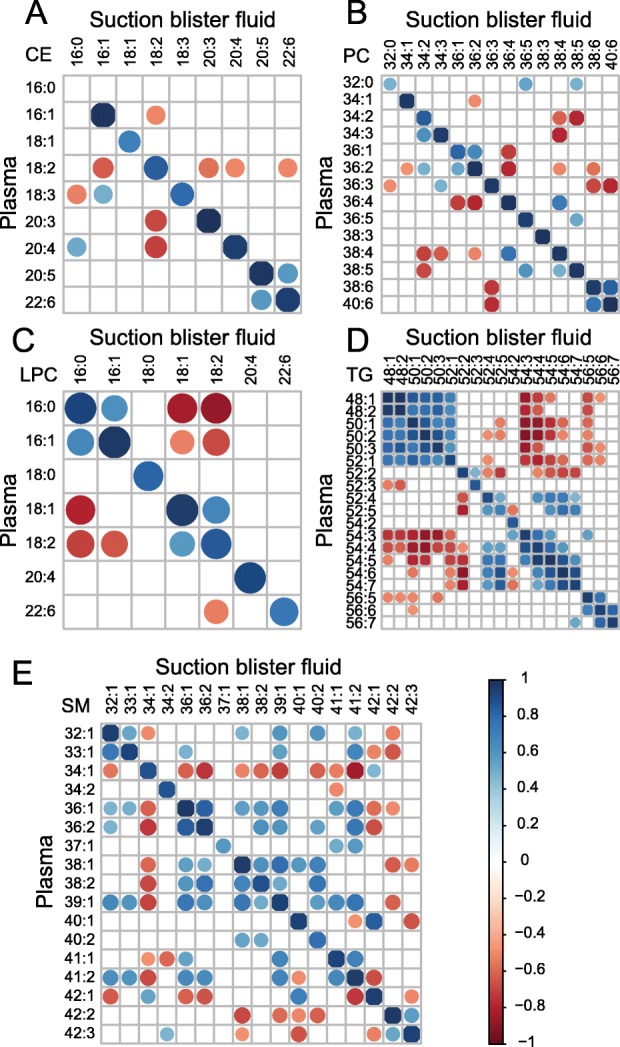
Correlation matrices for lipid species quantified in plasma and SBF. The molar percent of lipid species in cholesteryl esters (CE) **a**, phosphatidylcholine (PC) **b**, lysophosphatidylcholine (LPC) **c**, triglycerides (TG) **d**, and sphingomyelins (SM) (**e**) comparing fractions in plasma (vertical) to SBF (horizontal). The size and color of the dots are proportional to the Pearson correlation coefficient. Only significant correlations (*p* < 0.05) are shown. *n* = 18

To investigate the relationship between lipid species in the two fluids further, correlation analyses between plasma and SBF were performed for all the lipid species found above 1 molar percent (Fig. 4). For CEs, all plasma lipid species were significantly correlated with their SBF counterparts (Pearson’s correlation coefficient, *p* < 0.05), with the exception of cholesteryl palmitate (CE16:0) (Fig. 4a). However, cholesteryl palmitate was the only CE that was not normally distributed. When the non-parametric Spearman’s rho test was applied, this lipid species also showed a significant correlation between plasma and SBF (*ρ* = 0.589, *p* = 0.01). Lipid species in plasma within the classes LPC, PC, TG, and SM were positively and significantly correlated with their SBF counterparts (Fig. 4b-e).

### Inter- and intravariability of lipid species in plasma and suction blister fluid

**Fig. 5:**
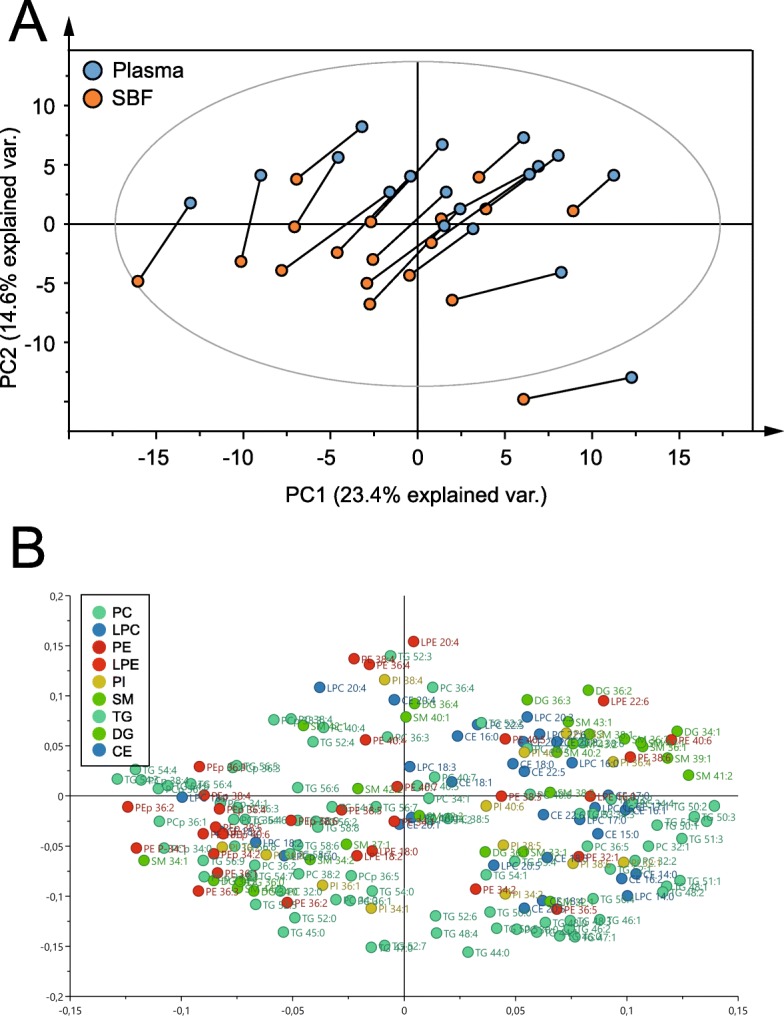
Unsupervised separation of plasma and SBF samples. Samples were separated based on molar percent of lipids using a principal component analysis (PCA). PCA score plot (**a**) and corresponding loading scatter plot (**b**) (*n* = 18 × 2). Samples from the same subject are connected with a line in the PCA plot. The model included 191 lipid variables. The ellipse in A shows Hotelling’s T^2^ (95% confidence limit). Lipid species are colored according to lipid class as shown in the legend. CE, cholesteryl ester; DG, diglyceride; LPC, lysophosphatidylcholine; LPE, lysophosphatidylethanolamine; PC, phosphatidylcholine; PE, phosphatidylethanolamine; PI, phosphatidylinositol; SM, sphingomyelin; TG, triglyceride

**Fig. 6:**
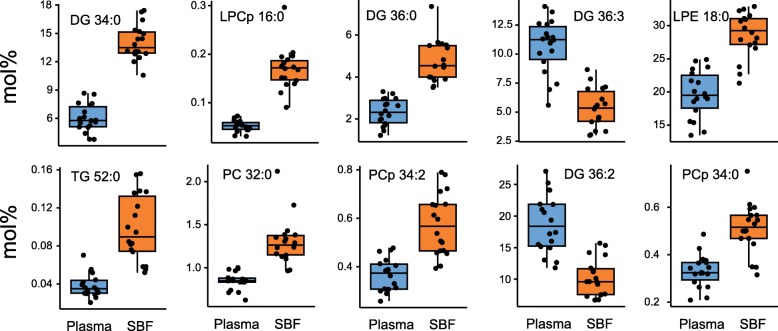
Comparison of molar fractions of the 10 lipid species with the highest VIP scores. VIP scores were retrieved from an OPLS-DA analysis that aimed to discriminate plasma from SBF samples. A high VIP score indicates high ability to discriminate samples according to the OPLS-DA model. All 10 lipid species showed significant differences between plasma and SBF (Mann-Whitney *U* test, *p* < 0.001)

Next, we examined how the proportions of lipid species differed between and within study subjects (i.e., between plasma and SBF). To this end, the molar fraction of all analyzed lipids excluding PS (because this group of lipids was only represented by two molecular species) in all participants were used in a principal component analysis (PCA). This yielded a seven-component PCA (R^2^X = 0.770 [goodness of fit], Q^2^ = 0.463 [goodness of prediction]) with 23.4 and 14.6% of the variance explained by the first and second principal component, respectively (Fig. 5a and corresponding loading plot Fig. 5b). Plasma and SBF samples from the same individual are connected with a line in Fig. 5a to illustrate variability between and within subjects. Plasma and SBF samples separated similarly for all participants; this manifested as a shift from top right to bottom left in the PCA score plot, apparently dependent on both the first and second components. The sample distance was greater between subjects than within subjects for plasma and SBF, indicating that the interindividual variation was larger than the intraindividual variation. The uniform separation of plasma and SBF samples within subjects suggested common lipid composition differences. To test whether plasma and SBF samples could be statistically separated based on lipid composition, an orthogonal projection to latent structures discriminant analysis (OPLS-DA) was performed. The OPLS-DA model scatterplot separates samples according to group classification (i.e., plasma or SBF) on the x-axis and captures other within-group variations vertically (OPLS-DA model 1 [predictive] + 4 [orthogonal] components, R^2^X = 0.589, R^2^Y = 0.982, Q^2^ = 0.899, Additional file 2: Figure S2A). The corresponding loading scatter plot displays the relation between the X-variables and Y-variables for sample class and the first Y-orthogonal component (Additional file 2: Figure S2B). The ability of the OPLS-DA model to discriminate plasma from SBF samples was validated by a permutation test in which sample class was randomly permuted 999 times (Additional file 2: Figure S2C). R^2^Y and Q^2^ values for all permutated models were lower than for the original model. This test indicated that the model was able to adequately predict sample class significantly better than chance. Further, the statistical significance of the model was confirmed by a cross validation ANOVA test (CV-ANOVA, *p* = 5.0 · 10^− 10^). Notably, in the OPLS-DA score plot (Additional file 2: Figure S2A), the variation was greater in the Y direction than in the X direction, again pointing to interindividual variation exceeding intraindividual variation. Figure 6 shows a comparison between plasma and SBF of the 10 lipid species with the greatest importance for explaining discrimination of sample class in the OPLS-DA model (VIP scores, Variable Importance for the Projection). There were statistically significant differences between biofluids for all 10 of these lipid species (Mann-Whitney *U* test, *p* < 0.001). Interestingly, among these 10 lipid species, four were DGs, indicating that the composition of this lipid class differs particularly between plasma and SBF. The remaining six compounds included two lysophospholipid species, 3 PC species (of which two where plasmalogens), and one TG species. A complete list of VIP scores and group comparisons between plasma and SBF for the analyzed lipid species is provided in Additional file 6: Table S3.

### Fatty acids in plasma and suction blister fluid

**Fig. 7:**
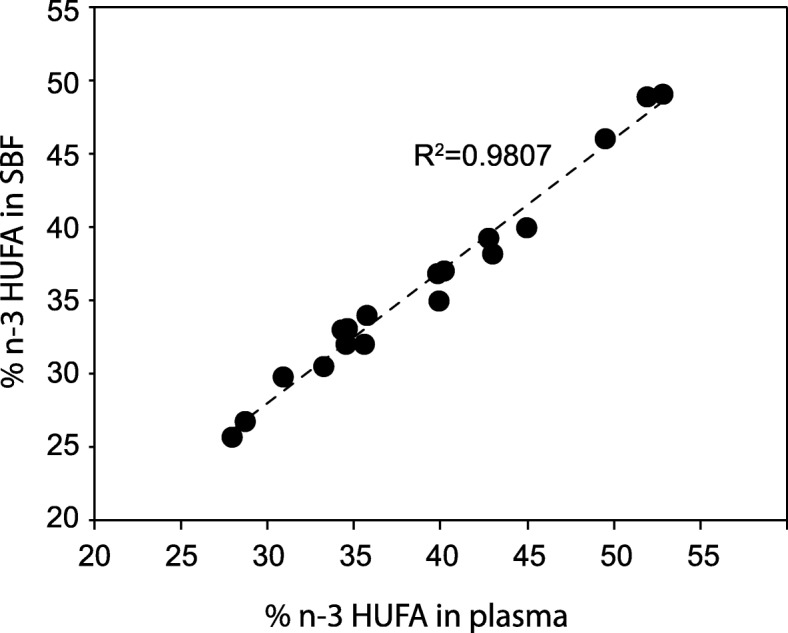
Fatty acids in plasma and SBF. A scatter plot showing the association between the fraction of n-3 highly unsaturated fatty acids (HUFAs) in plasma and SBF

Next, we determined the total fatty acid profiles of plasma and SBF through GC-MS analysis of FAMEs. In accordance with the other data, fatty acid composition in plasma and SBF were similar and highly correlated (Additional file 3: Figure S3A and B). The proportion of n-3 highly unsaturated fatty acids (20–22 carbons with at least three double bonds, HUFAs) was strongly correlated between plasma and SBF (Fig. 7). However, plasma had a slightly larger fraction of n-3 HUFAs than SBF.

## Discussion

The development of non-invasive and painless techniques to extract ISF has spurred a growing interest in analyzing protein and metabolite levels in this hitherto overlooked sample matrix. Despite this, biomarkers found in ISF that could be used for clinical monitoring are poorly explored. We determined the composition of lipid species and total fatty acid profiles of SBF as a surrogate to ISF, and compared that to plasma. Our results conclusively show that plasma and SBF have very similar lipid composition, and that SBF, or pure interstitial fluid, may be used interchangeably with blood when screening for many pathophysiological lipid markers.

The total concentration of quantified lipids was approximately five-fold higher in plasma compared to SBF. This is in agreement with previous data on lipid fractions in human blister fluid [17, 19]. While the overall lipid profiles of plasma and SBF were similar, there were small yet significant differences in both lipid class and species composition. On a lipid class level, SBF had a larger fraction of lysophospholipids (LPC and LPE) and DG than plasma. These point to a higher release of fatty acid esters from PC/PE and TG in SBF compared to plasma. Indeed, fractions of PC, PE, and TG were significantly lower in SBF. Although levels of NEFAs were not determined in this study, LC-MS scan data from pooled SBF versus plasma samples lend support to the notion that NEFAs are more abundant in SBF. Furthermore, plasma and SBF samples could successfully be discriminated with an OPLS-DA model, and a subsequent VIP analysis identified several DG and LPC species that were significantly under- or overrepresented in SBF. Our analyses cannot exclude that the differences in lipid profiles between plasma and SBF are artefacts of the fluid collection or lipid extraction (i.e., that lipolysis is stimulated by the blister formation per se), or that lipids are metabolized to a greater extent during extraction from SBF compared to plasma. Future studies should aim to resolve these questions using microneedle systems to extract pure dermal interstitial fluid.

A recent report addressed the relationship of bioactive lipid mediators, including prostanoids, endocannabinoids and hydroxy fatty acids, in different dermal cell layers and SBF [6]. The authors found that SBF lipid profile was similar to that of epidermis, indicating that bioactive lipids in SBF are primarily of epidermal origin. It is thus plausible that the differences in lipid profiles reported herein between plasma and SBF can be attributed to the influence of dermal cell layers.

All plasma lipid species found at concentrations greater than 1 molar percent within the major lipid classes correlated positively with their SBF counterpart, with Pearson correlation coefficients generally > 0.8. Apart from direct relationships between the same lipid species in the two compartments, the correlation analysis also revealed significant associations between other lipid species. For example, lipids containing 16:1 fatty acid moieties correlated negatively with lipids containing 18:2 fatty acids (compare CE 16:1/18:2, LPC 16:1/18:2, PC 34:1/36:2, SM 34:1/36:2, TG 48:1/54:3 and FA 16:1 n-7/18:1 n-9). There was high consistency in correlation patterns between *plasma* vs. *SBF* and *SBF* vs. *plasma*, indicating high robustness in our analyses.

Although LC-MS/MS analysis provides extensive information about lipid species composition in terms of hydrocarbon chain length and degree of unsaturation, location of the double bonds within acyl chains is difficult to resolve. We used GC-MS to determine the fatty acid composition; in particular, we analyzed the relationship between n-3 fatty acids in plasma and SBF. There have been several proposed biomarkers for monitoring blood and tissue n-3 status [36–38]. One of the most consistent in terms of low experimental noise and high correlations between blood fractions is the HUFA-based approach [39]. Although plasma was found to have a slightly larger fraction of n-3 HUFAs than SBF, the correlation between the two fluids was nearly complete (*R*^*2*^ = 0.98). Furthermore, all LC-PUFAs found at greater than 1 molar percent displayed excellent correlations between plasma and SBF (*R*^*2*^ > 0.96). LC-PUFAs, including docosahexaenoic acid (DHA, 22:6 n-3) and arachidonic acid (AA, 20:4 n-6), are vital for eye and neural development. These fatty acids are transported in a selective manner from the mother to the growing fetus during pregnancy [40, 41]. In the instance of very or extremely preterm birth, this transfer is abruptly interrupted before completion, with consequences for LC-PUFA accretion in the infant brain, eyes, and other organs [42]. There is growing evidence that supplementation of LC-PUFAs can partly restore infant fatty acid stores and limit certain morbidities associated with prematurity [43–45]. Because extensive blood sampling in this group of fragile infants may lead to anemia and further promote disease [46], ISF may potentially be used as a replacement for blood to monitor postnatal LC-PUFA status and provide grounds for deciding on a personalized supplementation strategy.

## Conclusions

We report that the interindividual variation in SBF lipid profiles is much larger than the within-subject variation between plasma and SBF, and that SBF can be used in place of blood to determine the status of lipid biomarkers. The development of microneedle systems to extract dermal ISF will allow the utilization of this biofluid in health monitoring.

## Additional files

Additional file 1: **Figure S1.** Relative abundance of lipids in plasma and SBF. The molar percent of lipid species found within lipid classes diglycerides (DG) (**A**), lysophosphatidylethanolamines (LPE) (**B**), phosphatidylethanolamines (PE) (**C**), and phosphatidylinositols (PI) (**D**). Only lipid species exceeding one molar percent are shown. Mean and SD are shown (*n* = 18). (EPS 2620 kb)

Additional file 2: **Figure S2.** OPLS-DA model based on the molar percentage of lipids in plasma and SBF. OPLS-DA score scatter plot (**A**) and corresponding loading scatter plot (**B**) where *n* = 18 × 2. Samples are separated based on class (i.e., plasma or SBF). Lipid species are colored according to lipid class as shown in the legend. (**C**) Evaluation of the OPLS-DA model with a permutation test where sample class was randomly permuted (*n* = 999). The vertical axis in the plot shows values of R^2^ and Q^2^ for the permuted models (*left*) and for the original model (*far right*). The horizontal axis shows the correlation between the permuted sample class and the original sample class. CE, cholesteryl ester; DG, diglyceride; LPC, lysophosphatidylcholine; LPE, lysophosphatidylethanolamine; PC, phosphatidylcholine; PE, phosphatidylethanolamine; PI, phosphatidylinositol; SM, sphingomyelin; TG, triglyceride. (EPS 1970 kb)

Additional file 3: **Figure S3**. Comparison of total fatty acids in plasma and SBF. (**A**) Molar fraction of the most abundant fatty acids in plasma and SBF. Mean and SD are shown (n = 18). (**B**) Correlation matrix for fatty acid in plasma and SBF. The size and color of the dots are proportional to the Pearson correlation coefficient. Only significant correlations (*p* < 0.05) are shown (n = 18). (EPS 2920 kb)

Additional file 4: **Table S1.** Precursor and product ions used for multiple reaction monitoring (MRM). (DOCX 48 kb)

Additional file 5: **Table S2.** Molar fraction and concentration of quantified lipids in plasma and SBF. Molar fractions represent the percentage of individual lipid species within their lipid class. *n* = 18. (DOCX 65 kb)

Additional file 6: **Table S3.** Predictive variable importance for the OPLS-DA projection (VIP score) and group comparison of lipid species in plasma and SBF using the Mann–Whitney *U* test. (DOCX 29 kb)

## Data Availability

The datasets used and analyzed during the current study are available from the corresponding author on reasonable request.

## Abbreviations

CE: Cholesteryl ester
HUFA: Highly unsaturated fatty acids
ISF: Interstitial fluid
LC-PUFA: Long-chain polyunsaturated fatty acids
PCp: PC plasmalogen
PEp: PE plasmalogen
SBF: Suction blister fluid
