# Supplementary material for: Lipid profiling of suction blister fluid: comparison of lipids in interstitial fluid and plasma
Source: Lipids Health Dis. 2019 Aug 24;18:164. doi: 10.1186/s12944-019-1107-3 (PMC6708155; doi:10.1186/s12944-019-1107-3)
Supplement: Supplementary file 4 — Table S1. Precursor and product ions used for multiple reaction monitoring (MRM). (DOCX 48 kb) [file 12944_2019_1107_MOESM4_ESM.docx]

**Additional file 4: Table S1: Precursor and product ions used for multiple reaction monitoring (MRM).**

| Lipid class | Lipid species | Parent Ion | Precursor Ion (Q1) | Product Ion (Q3) | Type |
| --- | --- | --- | --- | --- | --- |
| LPC | LPC 14:0 | [M+H]^+^ | 468.3 | 184.1 | Target |
| LPC | LPC 15:0 | [M+H]^+^ | 482.3 | 184.1 | Target |
| LPC | LPC 16:0 | [M+H]^+^ | 496.3 | 184.1 | Target |
| LPC (plasmalogen) | LPCp 16:0 | [M+H]^+^ | 480.3 | 184.1 | Target |
| LPC | LPC 16:1 | [M+H]^+^ | 494.3 | 184.1 | Target |
| LPC | LPC 17:0 | [M+H]^+^ | 510.4 | 184.1 | Target |
| LPC | LPC 18:0 | [M+H]^+^ | 524.4 | 184.1 | Target |
| LPC | LPC 18:1 | [M+H]^+^ | 522.4 | 184.1 | Target |
| LPC | LPC 18:1(d7) | [M+H]^+^ | 529.4 | 184.1 | IS |
| LPC | LPC 18:2 | [M+H]^+^ | 520.3 | 184.1 | Target |
| LPC | LPC 18:3 | [M+H]^+^ | 518.3 | 184.1 | Target |
| LPC | LPC 20:3 | [M+H]^+^ | 546.3 | 184.1 | Target |
| LPC | LPC 20:4 | [M+H]^+^ | 544.3 | 184.1 | Target |
| LPC | LPC 20:5 | [M+H]^+^ | 542.3 | 184.1 | Target |
| LPC | LPC 22:5 | [M+H]^+^ | 570.3 | 184.1 | Target |
| LPC | LPC 22:6 | [M+H]^+^ | 568.3 | 184.1 | Target |
| PC | PC 30:0 | [M+H]^+^ | 706.5 | 184.1 | Target |
| PC | PC 32:0 | [M+H]^+^ | 734.6 | 184.1 | Target |
| PC | PC 32:1 | [M+H]^+^ | 732.5 | 184.1 | Target |
| PC | PC 32:2 | [M+H]^+^ | 730.5 | 184.1 | Target |
| PC | PC 33:1 \| 15:0/18:1(d7) | [M+H]^+^ | 753.6 | 184.1 | IS |
| PC | PC 34:0 | [M+H]^+^ | 762.6 | 184.1 | Target |
| PC | PC 34:1 | [M+H]^+^ | 760.6 | 184.1 | Target |
| PC | PC 34:2 | [M+H]^+^ | 758.6 | 184.1 | Target |
| PC | PC 34:3 | [M+H]^+^ | 756.6 | 184.1 | Target |
| PC | PC 34:4 | [M+H]^+^ | 754.5 | 184.1 | Target |
| PC | PC 36:1 | [M+H]^+^ | 788.6 | 184.1 | Target |
| PC | PC 36:2 | [M+H]^+^ | 786.6 | 184.1 | Target |
| PC | PC 36:3 | [M+H]^+^ | 784.6 | 184.1 | Target |
| PC | PC 36:4 | [M+H]^+^ | 782.6 | 184.1 | Target |
| PC | PC 36:5 | [M+H]^+^ | 780.6 | 184.1 | Target |
| PC | PC 38:2 | [M+H]^+^ | 814.6 | 184.1 | Target |
| PC | PC 38:3 | [M+H]^+^ | 812.6 | 184.1 | Target |
| PC | PC 38:4 | [M+H]^+^ | 810.6 | 184.1 | Target |
| PC | PC 38:5 | [M+H]^+^ | 808.6 | 184.1 | Target |
| PC | PC 38:6 | [M+H]^+^ | 806.6 | 184.1 | Target |
| PC | PC 40:4 | [M+H]^+^ | 838.6 | 184.1 | Target |
| PC | PC 40:5 | [M+H]^+^ | 836.6 | 184.1 | Target |
| PC | PC 40:6 | [M+H]^+^ | 834.6 | 184.1 | Target |
| PC | PC 40:7 | [M+H]^+^ | 832.6 | 184.1 | Target |
| PC (plasmalogen) | PCp 34:0 | [M+H]^+^ | 746.6 | 184.1 | Target |
| PC (plasmalogen) | PCp 34:1 | [M+H]^+^ | 744.6 | 184.1 | Target |
| PC (plasmalogen) | PCp 34:2 | [M+H]^+^ | 742.6 | 184.1 | Target |
| PC (plasmalogen) | PCp 36:1 | [M+H]^+^ | 772.6 | 184.1 | Target |
| PC (plasmalogen) | PCp 36:2 | [M+H]^+^ | 770.6 | 184.1 | Target |
| PC (plasmalogen) | PCp 36:1 \| P-18:0/18:1(d9) | [M+H]^+^ | 781.7 | 184.1 | IS |
| PC (plasmalogen) | PCp 36:3 | [M+H]^+^ | 768.6 | 184.1 | Target |
| PC (plasmalogen) | PCp 36:4 | [M+H]^+^ | 766.6 | 184.1 | Target |
| PC (plasmalogen) | PCp 36:5 | [M+H]^+^ | 764.6 | 184.1 | Target |
| PC (plasmalogen) | PCp 38:3 | [M+H]^+^ | 796.6 | 184.1 | Target |
| PC (plasmalogen) | PC 38:4 | [M+H]^+^ | 794.6 | 184.1 | Target |
| LPE | LPE 16:0 | [M+H]^+^ | 454.3 | 313.3 | Target |
| LPE | LPE 18:0 | [M+H]^+^ | 482.3 | 341.3 | Target |
| LPE | LPE 18:1 | [M+H]^+^ | 480.3 | 339.3 | Target |
| LPE | LPE 18:1(d7) | [M+H]^+^ | 487.3 | 346.3 | IS |
| LPE | LPE 18:2 | [M+H]^+^ | 478.3 | 337.3 | Target |
| LPE | LPE 20:4 | [M+H]^+^ | 502.3 | 361.3 | Target |
| LPE | LPE 22:6 | [M+H]^+^ | 526.3 | 385.3 | Target |
| PE | PE 32:1 | [M+H]^+^ | 690.5 | 549.5 | Target |
| PE | PE 33:1 \| 15:0/18:1 (d7) | [M+H]^+^ | 711.5 | 570.5 | IS |
| PE | PE 34:0 | [M+H]^+^ | 720.6 | 579.6 | Target |
| PE | PE 34:1 | [M+H]^+^ | 718.5 | 577.5 | Target |
| PE | PE 34:2 | [M+H]^+^ | 716.5 | 575.5 | Target |
| PE | PE 36:1 | [M+H]^+^ | 746.6 | 605.6 | Target |
| PE | PE 36:2 | [M+H]^+^ | 744.6 | 603.6 | Target |
| PE | PE 36:3 | [M+H]^+^ | 742.5 | 601.5 | Target |
| PE | PE 36:4 | [M+H]^+^ | 740.5 | 599.5 | Target |
| PE | PE 36:5 | [M+H]^+^ | 738.5 | 597.5 | Target |
| PE | PE 38:1 | [M+H]^+^ | 774.6 | 633.6 | Target |
| PE | PE 38:2 | [M+H]^+^ | 772.6 | 631.6 | Target |
| PE | PE 38:3 | [M+H]^+^ | 770.6 | 629.6 | Target |
| PE | PE 38:4 | [M+H]^+^ | 768.6 | 627.6 | Target |
| PE | PE 38:5 | [M+H]^+^ | 766.5 | 625.5 | Target |
| PE | PE 38:6 | [M+H]^+^ | 764.5 | 623.5 | Target |
| PE | PE 40:4 | [M+H]^+^ | 796.6 | 655.6 | Target |
| PE | PE 40:5 | [M+H]^+^ | 794.6 | 653.6 | Target |
| PE | PE 40:6 | [M+H]^+^ | 792.6 | 651.6 | Target |
| PE | PE 40:7 | [M+H]^+^ | 790.5 | 649.5 | Target |
| PE (plasmalogen) | PEp 34:1 | [M+H]^+^ | 702.5 | 561.5 | Target |
| PE (plasmalogen) | PEp 34:2 | [M+H]^+^ | 700.5 | 559.5 | Target |
| PE (plasmalogen) | PEp 36:1 \| P-18:0/18:1 (d9) | [M+H]^+^ | 730.5 | 589.5 | Target |
| PE (plasmalogen) | PEp 36:1 | [M+H]^+^ | 739.6 | 598.6 | IS |
| PE (plasmalogen) | PEp 36:2 | [M+H]^+^ | 728.5 | 587.5 | Target |
| PE (plasmalogen) | PEp 36:3 | [M+H]^+^ | 726.5 | 585.5 | Target |
| PE (plasmalogen) | PEp 36:4 | [M+H]^+^ | 724.5 | 583.5 | Target |
| PE (plasmalogen) | PEp 38:3 | [M+H]^+^ | 754.5 | 613.5 | Target |
| PE (plasmalogen) | PEp 38:4 | [M+H]^+^ | 752.5 | 611.5 | Target |
| PE (plasmalogen) | PEp 38:5 | [M+H]^+^ | 750.5 | 609.5 | Target |
| PE (plasmalogen) | PEp 38:6 | [M+H]^+^ | 748.6 | 607.6 | Target |
| PE (plasmalogen) | PEp 40:4 | [M+H]^+^ | 780.6 | 639.6 | Target |
| PE (plasmalogen) | PEp 40:5 | [M+H]^+^ | 778.5 | 637.5 | Target |
| PE (plasmalogen) | PEp 40:6 | [M+H]^+^ | 776.5 | 635.5 | Target |
| PI | PI 32:1 | [M+H]^+^ | 809.5 | 549.5 | Target |
| PI | PI 33:1 \| 15:0/18:1(d7) | [M+H]^+^ | 830.5 | 570.5 | IS |
| PI | PI 34:3 | [M+H]^+^ | 833.5 | 573.5 | Target |
| PI | PI 34:2 | [M+H]^+^ | 835.5 | 575.5 | Target |
| PI | PI 34:1 | [M+H]^+^ | 837.5 | 577.5 | Target |
| PI | PI 36:4 | [M+H]^+^ | 859.5 | 599.5 | Target |
| PI | PI 36:3 | [M+H]^+^ | 861.5 | 601.5 | Target |
| PI | PI 36:2 | [M+H]^+^ | 863.6 | 603.6 | Target |
| PI | PI 36:1 | [M+H]^+^ | 865.6 | 605.6 | Target |
| PI | PI 38:3 | [M+H]^+^ | 889.6 | 629.6 | Target |
| PI | PI 38:4 | [M+H]^+^ | 887.5 | 627.5 | Target |
| PI | PI 38:5 | [M+H]^+^ | 885.5 | 625.5 | Target |
| PI | PI 38:6 | [M+H]^+^ | 883.5 | 623.5 | Target |
| PI | PI 40:4 | [M+H]^+^ | 915.6 | 655.6 | Target |
| PI | PI 40:5 | [M+H]^+^ | 913.6 | 653.6 | Target |
| PI | PI 40:6 | [M+H]^+^ | 911.6 | 651.6 | Target |
| PS | PS 33:1 \| 15:0/18:1(d7) | [M+H]^+^ | 755.5 | 570.5 | IS |
| PS | PS 34:1 | [M+H]^+^ | 762.5 | 577.5 | Target |
| PS | PS 36:1 | [M+H]^+^ | 790.6 | 605.6 | Target |
| PS | PS 36:2 | [M+H]^+^ | 788.5 | 603.5 | Target |
| PS | PS 36:4 | [M+H]^+^ | 784.5 | 599.5 | Target |
| PS | PS 38:1 | [M+H]^+^ | 818.6 | 633.6 | Target |
| PS | PS 38:4 | [M+H]^+^ | 812.5 | 627.5 | Target |
| PS | PS 38:5 | [M+H]^+^ | 810.5 | 625.5 | Target |
| PS | PS 40:4 | [M+H]^+^ | 840.5 | 655.5 | Target |
| PS | PS 40:5 | [M+H]^+^ | 838.5 | 653.5 | Target |
| PS | PS 40:6 | [M+H]^+^ | 836.5 | 651.5 | Target |
| SM | SM 32:1 | [M+H]^+^ | 675.5 | 184.1 | Target |
| SM | SM 33:1 | [M+H]^+^ | 689.6 | 184.1 | Target |
| SM | SM 34:1 | [M+H]^+^ | 703.6 | 184.1 | Target |
| SM | SM 34:2 | [M+H]^+^ | 701.6 | 184.1 | Target |
| SM | SM 36:1 | [M+H]^+^ | 731.6 | 184.1 | Target |
| SM | SM 36:2 | [M+H]^+^ | 729.6 | 184.1 | Target |
| SM | SM 36:2 \| 18:1/18:1(d9) | [M+H]^+^ | 738.6 | 184.1 | IS |
| SM | SM 37:1 | [M+H]^+^ | 745.6 | 184.1 | Target |
| SM | SM 38:1 | [M+H]^+^ | 759.6 | 184.1 | Target |
| SM | SM 38:2 | [M+H]^+^ | 757.6 | 184.1 | Target |
| SM | SM 39:1 | [M+H]^+^ | 773.7 | 184.1 | Target |
| SM | SM 40:1 | [M+H]^+^ | 787.7 | 184.1 | Target |
| SM | SM 40:2 | [M+H]^+^ | 785.7 | 184.1 | Target |
| SM | SM 41:1 | [M+H]^+^ | 801.7 | 184.1 | Target |
| SM | SM 41:2 | [M+H]^+^ | 799.7 | 184.1 | Target |
| SM | SM 42:1 | [M+H]^+^ | 815.7 | 184.1 | Target |
| SM | SM 42:2 | [M+H]^+^ | 813.7 | 184.1 | Target |
| SM | SM 42:3 | [M+H]^+^ | 811.7 | 184.1 | Target |
| SM | SM 43:1 | [M+H]^+^ | 829.7 | 184.1 | Target |
| SM | SM 43:2 | [M+H]^+^ | 827.7 | 184.1 | Target |
| SM | SM 44:2 | [M+H]^+^ | 841.7 | 184.1 | Target |
| SM | SM 44:1 | [M+H]^+^ | 843.7 | 184.1 | Target |
| DG | DG 30:0 | [M+NH4]^+^ | 558.5 | 558.5 | Target |
| DG | DG 30:1 | [M+NH4]^+^ | 556.5 | 556.5 | Target |
| DG | DG 30:2 | [M+NH4]^+^ | 554.5 | 554.5 | Target |
| DG | DG 32:0 | [M+NH4]^+^ | 586.5 | 586.5 | Target |
| DG | DG 32:1 | [M+NH4]^+^ | 584.5 | 584.5 | Target |
| DG | DG 32:2 | [M+NH4]^+^ | 582.5 | 582.5 | Target |
| DG | DG 32:3 | [M+NH4]^+^ | 580.5 | 580.5 | Target |
| DG | DG:33:1 \| 15:0/18:1(d7) | [M+NH4]^+^ | 605.5 | 605.5 | IS |
| DG | DG 34:0 | [M+NH4]^+^ | 614.6 | 614.6 | Target |
| DG | DG 34:1 | [M+NH4]^+^ | 612.6 | 612.6 | Target |
| DG | DG 34:2 | [M+NH4]^+^ | 610.5 | 610.5 | Target |
| DG | DG 34:3 | [M+NH4]^+^ | 608.5 | 608.5 | Target |
| DG | DG 34:4 | [M+NH4]^+^ | 606.5 | 606.5 | Target |
| DG | DG 36:0 | [M+NH4]^+^ | 642.6 | 642.6 | Target |
| DG | DG 36:1 | [M+NH4]^+^ | 640.6 | 640.6 | Target |
| DG | DG 36:2 | [M+NH4]^+^ | 638.6 | 638.6 | Target |
| DG | DG 36:3 | [M+NH4]^+^ | 636.5 | 636.5 | Target |
| DG | DG 36:4 | [M+NH4]^+^ | 634.5 | 634.5 | Target |
| DG | DG 36:5 | [M+NH4]^+^ | 632.5 | 632.5 | Target |
| DG | DG 38:0 | [M+NH4]^+^ | 670.6 | 670.6 | Target |
| DG | DG 38:1 | [M+NH4]^+^ | 668.6 | 668.6 | Target |
| DG | DG 38:2 | [M+NH4]^+^ | 666.6 | 666.6 | Target |
| DG | DG 38:3 | [M+NH4]^+^ | 664.6 | 664.6 | Target |
| DG | DG 38:4 | [M+NH4]^+^ | 662.6 | 662.6 | Target |
| DG | DG 38:5 | [M+NH4]^+^ | 660.6 | 660.6 | Target |
| DG | DG 38:6 | [M+NH4]^+^ | 658.5 | 658.5 | Target |
| DG | DG 40:4 | [M+NH4]^+^ | 690.6 | 690.6 | Target |
| DG | DG 40:5 | [M+NH4]^+^ | 688.6 | 688.6 | Target |
| DG | DG 40:6 | [M+NH4]^+^ | 686.6 | 686.6 | Target |
| DG | DG 40:7 | [M+NH4]^+^ | 684.6 | 684.6 | Target |
| TG | TG 44:1 | [M+NH4]^+^ | 766.7 | 766.7 | Target |
| TG | TG 44:0 | [M+NH4]^+^ | 768.7 | 768.7 | Target |
| TG | TG 45:0 | [M+NH4]^+^ | 782.7 | 782.7 | Target |
| TG | TG 46:2 | [M+NH4]^+^ | 792.7 | 792.7 | Target |
| TG | TG 46:1 | [M+NH4]^+^ | 794.7 | 794.7 | Target |
| TG | TG 46:0 | [M+NH4]^+^ | 796.7 | 796.7 | Target |
| TG | TG 47:1 | [M+NH4]^+^ | 808.8 | 808.8 | Target |
| TG | TG 47:0 | [M+NH4]^+^ | 810.8 | 810.8 | Target |
| TG | TG 48:1 \| 15:0-18:1(d7)-15:0 | [M+NH4]^+^ | 829.8 | 829.8 | IS |
| TG | TG 48:4 | [M+NH4]^+^ | 816.7 | 816.7 | Target |
| TG | TG 48:3 | [M+NH4]^+^ | 818.7 | 818.7 | Target |
| TG | TG 48:2 | [M+NH4]^+^ | 820.7 | 820.7 | Target |
| TG | TG 48:1 | [M+NH4]^+^ | 822.8 | 822.7 | Target |
| TG | TG 48:0 | [M+NH4]^+^ | 824.8 | 824.7 | Target |
| TG | TG 49:3 | [M+NH4]^+^ | 832.7 | 832.7 | Target |
| TG | TG 49:2 | [M+NH4]^+^ | 834.8 | 834.8 | Target |
| TG | TG 49:1 | [M+NH4]^+^ | 836.8 | 836.8 | Target |
| TG | TG 50:5 | [M+NH4]^+^ | 842.7 | 842.6 | Target |
| TG | TG 50:4 | [M+NH4]^+^ | 844.7 | 844.6 | Target |
| TG | TG 50:3 | [M+NH4]^+^ | 846.7 | 846.6 | Target |
| TG | TG 50:2 | [M+NH4]^+^ | 848.8 | 848.6 | Target |
| TG | TG 50:1 | [M+NH4]^+^ | 850.7 | 850.7 | Target |
| TG | TG 50:0 | [M+NH4]^+^ | 852.8 | 852.8 | Target |
| TG | TG 51:3 | [M+NH4]^+^ | 860.8 | 860.8 | Target |
| TG | TG 51:2 | [M+NH4]^+^ | 862.8 | 862.8 | Target |
| TG | TG 51:1 | [M+NH4]^+^ | 864.8 | 864.8 | Target |
| TG | TG 52:7 | [M+NH4]^+^ | 866.8 | 866.8 | Target |
| TG | TG 52:6 | [M+NH4]^+^ | 868.8 | 868.8 | Target |
| TG | TG 52:5 | [M+NH4]^+^ | 870.8 | 870.8 | Target |
| TG | TG 52:4 | [M+NH4]^+^ | 872.8 | 872.8 | Target |
| TG | TG 52:3 | [M+NH4]^+^ | 874.8 | 874.8 | Target |
| TG | TG 52:2 | [M+NH4]^+^ | 876.8 | 876.8 | Target |
| TG | TG 52:1 | [M+NH4]^+^ | 878.8 | 878.8 | Target |
| TG | TG 52:0 | [M+NH4]^+^ | 880.8 | 880.8 | Target |
| TG | TG 53:4 | [M+NH4]^+^ | 886.8 | 886.8 | Target |
| TG | TG 53:3 | [M+NH4]^+^ | 888.8 | 888.8 | Target |
| TG | TG 53:2 | [M+NH4]^+^ | 890.8 | 890.8 | Target |
| TG | TG 54:7 | [M+NH4]^+^ | 894.8 | 894.8 | Target |
| TG | TG 54:6 | [M+NH4]^+^ | 896.8 | 896.8 | Target |
| TG | TG 54:5 | [M+NH4]^+^ | 898.8 | 898.8 | Target |
| TG | TG 54:4 | [M+NH4]^+^ | 900.8 | 900.8 | Target |
| TG | TG 54:3 | [M+NH4]^+^ | 902.8 | 902.8 | Target |
| TG | TG 54:2 | [M+NH4]^+^ | 904.8 | 904.8 | Target |
| TG | TG 54:1 | [M+NH4]^+^ | 906.8 | 906.8 | Target |
| TG | TG 54:0 | [M+NH4]^+^ | 908.8 | 908.8 | Target |
| TG | TG 56:9 | [M+NH4]^+^ | 918.8 | 918.8 | Target |
| TG | TG 56:8 | [M+NH4]^+^ | 920.8 | 920.8 | Target |
| TG | TG 56:7 | [M+NH4]^+^ | 922.8 | 922.8 | Target |
| TG | TG 56:6 | [M+NH4]^+^ | 924.8 | 924.8 | Target |
| TG | TG 56:5 | [M+NH4]^+^ | 926.8 | 926.8 | Target |
| TG | TG 56:4 | [M+NH4]^+^ | 928.8 | 928.8 | Target |
| TG | TG 56:3 | [M+NH4]^+^ | 930.8 | 930.8 | Target |
| TG | TG 56:2 | [M+NH4]^+^ | 932.9 | 932.9 | Target |
| TG | TG 58:9 | [M+NH4]^+^ | 946.8 | 946.8 | Target |
| TG | TG 58:8 | [M+NH4]^+^ | 948.8 | 948.8 | Target |
| TG | TG 58:7 | [M+NH4]^+^ | 950.8 | 950.8 | Target |
| TG | TG 58:6 | [M+NH4]^+^ | 952.8 | 952.8 | Target |
| CE | CE 14:0 | [M+NH4]^+^ | 614.6 | 369.4 | Target |
| CE | CE 15:0 | [M+NH4]^+^ | 628.6 | 369.4 | Target |
| CE | CE 16:0 | [M+NH4]^+^ | 642.6 | 369.4 | Target |
| CE | CE 16:1 | [M+NH4]^+^ | 640.6 | 369.4 | Target |
| CE | CE 16:2 | [M+NH4]^+^ | 638.6 | 369.4 | Target |
| CE | CE 17:0 | [M+NH4]^+^ | 656.6 | 369.4 | Target |
| CE | CE 17:1 | [M+NH4]^+^ | 654.6 | 369.4 | Target |
| CE | CE 18:0 | [M+NH4]^+^ | 670.6 | 369.4 | Target |
| CE | CE 18:1 | [M+NH4]^+^ | 668.6 | 369.4 | Target |
| CE | CE 18:1(d7) | [M+NH4]^+^ | 675.6 | 369.4 | IS |
| CE | CE 18:2 | [M+NH4]^+^ | 666.6 | 369.4 | Target |
| CE | CE 18:3 | [M+NH4]^+^ | 664.6 | 369.4 | Target |
| CE | CE 18:4 | [M+NH4]^+^ | 662.6 | 369.4 | Target |
| CE | CE 20:1 | [M+NH4]^+^ | 696.7 | 369.4 | Target |
| CE | CE 20:2 | [M+NH4]^+^ | 694.6 | 369.4 | Target |
| CE | CE 20:3 | [M+NH4]^+^ | 692.6 | 369.4 | Target |
| CE | CE 20:4 | [M+NH4]^+^ | 690.6 | 369.4 | Target |
| CE | CE 20:5 | [M+NH4]^+^ | 688.6 | 369.4 | Target |
| CE | CE 22:5 | [M+NH4]^+^ | 716.6 | 369.4 | Target |
| CE | CE 22:6 | [M+NH4]^+^ | 714.6 | 369.4 | Target |
