# Supplementary material for: Lipid profiling of suction blister fluid: comparison of lipids in interstitial fluid and plasma
Source: Lipids Health Dis. 2019 Aug 24;18:164. doi: 10.1186/s12944-019-1107-3 (PMC6708155; doi:10.1186/s12944-019-1107-3)
Supplement: Supplementary file 5 — Table S2. Molar fraction and concentration of quantified lipids in plasma and SBF. Molar fractions represent the percentage of individual lipid species within their lipid class. n = 18. (DOCX 65 kb) [file 12944_2019_1107_MOESM5_ESM.docx]

**Additional file 5: Table S2: Molar fraction and concentration of quantified lipids in plasma and SBF. Molar fractions represent the percentage of individual lipid species within their lipid class. *n*=18.**

|  |  | Plasma | | SBF | | Plasma | | | SBF | | |
| --- | --- | --- | --- | --- | --- | --- | --- | --- | --- | --- | --- |
| Lipid class | **Lipid species** | **Mean (mol%)** | **SD (mol%)** | **Mean (mol%)** | **SD (mol%)** | **Mean (nmol/mL)** | **SD (nmol/mL)** | | **Mean (nmol/mL)** | **SD (nmol/mL)** | |
| Lyso phosphatidylcholine | LPC 14:0 | 0.62 | 0.25 | 0.764 | 0.276 | 1.09 | | 0.61 | 0.285 | | 0.114 |
| Lyso phosphatidylcholine | LPC 16:0 | 43.1 | 4.48 | 46 | 4.69 | 74.3 | | 21.1 | 17.1 | | 3.42 |
| Lyso phosphatidylcholine (plasmalogen) | LPCp 16:0 | 0.052 | 0.011 | 0.17 | 0.043 | 0.09 | | 0.029 | 0.062 | | 0.015 |
| Lyso phosphatidylcholine | LPC 16:1 | 1.09 | 0.301 | 1.21 | 0.269 | 1.86 | | 0.678 | 0.45 | | 0.121 |
| Lyso phosphatidylcholine | LPC 17:0 | 0.811 | 0.195 | 0.859 | 0.188 | 1.43 | | 0.613 | 0.327 | | 0.119 |
| Lyso phosphatidylcholine | LPC 18:0 | 15.9 | 2.25 | 16.4 | 1.64 | 28.3 | | 10.2 | 6.21 | | 1.62 |
| Lyso phosphatidylcholine | LPC 18:1 | 14.4 | 1.99 | 13.8 | 1.91 | 25.5 | | 8.78 | 5.2 | | 1.41 |
| Lyso phosphatidylcholine | LPC 18:2 | 18.2 | 3.77 | 16.2 | 3.68 | 32.4 | | 13.1 | 6.16 | | 2.07 |
| Lyso phosphatidylcholine | LPC 18:3 | 0.335 | 0.127 | 0.296 | 0.171 | 0.6 | | 0.322 | 0.106 | | 0.057 |
| Lyso phosphatidylcholine | LPC 20:3 | 1 | 0.346 | 0.734 | 0.2 | 1.65 | | 0.495 | 0.274 | | 0.086 |
| Lyso phosphatidylcholine | LPC 20:4 | 2.59 | 0.675 | 2.29 | 0.551 | 4.35 | | 1.43 | 0.848 | | 0.25 |
| Lyso phosphatidylcholine | LPC 20:5 | 0.576 | 0.212 | 0.484 | 0.181 | 1.02 | | 0.529 | 0.183 | | 0.084 |
| Lyso phosphatidylcholine | LPC 22:5 | 0.22 | 0.051 | 0.149 | 0.028 | 0.374 | | 0.116 | 0.056 | | 0.016 |
| Lyso phosphatidylcholine | LPC 22:6 | 1.01 | 0.275 | 0.709 | 0.17 | 1.68 | | 0.509 | 0.267 | | 0.094 |
| Phosphatidylcholine | PC 30:0 | 0.338 | 0.109 | 0.384 | 0.093 | 9.36 | | 4.93 | 1.56 | | 0.57 |
| Phosphatidylcholine | PC 32:0 | 0.849 | 0.102 | 1.3 | 0.271 | 22.4 | | 5.97 | 5.16 | | 1.31 |
| Phosphatidylcholine | PC 32:1 | 0.777 | 0.279 | 0.772 | 0.222 | 21.4 | | 10.7 | 3.21 | | 1.57 |
| Phosphatidylcholine | PC 32:2 | 0.24 | 0.111 | 0.171 | 0.064 | 6.84 | | 4.45 | 0.721 | | 0.4 |
| Phosphatidylcholine | PC 34:0 | 0.211 | 0.028 | 0.242 | 0.046 | 5.65 | | 1.88 | 0.96 | | 0.247 |
| Phosphatidylcholine | PC 34:1 | 14.2 | 1.07 | 14.6 | 1.19 | 376 | | 91.2 | 59 | | 17.4 |
| Phosphatidylcholine | PC 34:2 | 23.5 | 1.78 | 23.5 | 2.27 | 622 | | 160 | 95.3 | | 29 |
| Phosphatidylcholine | PC 34:3 | 1.05 | 0.279 | 0.787 | 0.219 | 28.6 | | 12.5 | 3.29 | | 1.59 |
| Phosphatidylcholine | PC 34:4 | 0.074 | 0.037 | 0.043 | 0.021 | 2.08 | | 1.37 | 0.182 | | 0.125 |
| Phosphatidylcholine | PC 36:1 | 2.91 | 0.52 | 3.04 | 0.437 | 78.1 | | 26.9 | 12.2 | | 3.36 |
| Phosphatidylcholine | PC 36:2 | 16 | 2.41 | 16.2 | 2.45 | 424 | | 127 | 64.8 | | 16.6 |
| Phosphatidylcholine | PC 36:3 | 8.88 | 0.822 | 8.39 | 0.784 | 234 | | 53.4 | 34 | | 9.79 |
| Phosphatidylcholine | PC 36:4 | 7.24 | 1.65 | 6.99 | 1.61 | 187 | | 51.3 | 28.3 | | 10.7 |
| Phosphatidylcholine | PC 36:5 | 2.06 | 0.769 | 1.49 | 0.495 | 56.2 | | 29.7 | 6.08 | | 2.69 |
| Phosphatidylcholine | PC 38:2 | 0.283 | 0.047 | 0.276 | 0.038 | 7.46 | | 2.19 | 1.1 | | 0.261 |
| Phosphatidylcholine | PC 38:3 | 1.88 | 0.56 | 1.8 | 0.555 | 49.1 | | 15.5 | 7.3 | | 3.03 |
| Phosphatidylcholine | PC 38:4 | 4.28 | 0.91 | 4.73 | 0.989 | 110 | | 24.5 | 18.8 | | 5.08 |
| Phosphatidylcholine | PC 38:5 | 2.94 | 0.415 | 2.9 | 0.391 | 77.3 | | 21.3 | 11.6 | | 2.88 |
| Phosphatidylcholine | PC 38:6 | 5.26 | 1.19 | 4.33 | 0.97 | 139 | | 46.2 | 17.8 | | 6.54 |
| Phosphatidylcholine | PC 40:4 | 0.122 | 0.028 | 0.13 | 0.031 | 3.15 | | 0.834 | 0.523 | | 0.176 |
| Phosphatidylcholine | PC 40:5 | 0.458 | 0.083 | 0.469 | 0.097 | 11.9 | | 3.03 | 1.87 | | 0.519 |
| Phosphatidylcholine | PC 40:6 | 2.96 | 0.673 | 2.84 | 0.644 | 78 | | 24.7 | 11.5 | | 3.53 |
| Phosphatidylcholine | PC 40:7 | 0.296 | 0.052 | 0.273 | 0.048 | 7.81 | | 2.29 | 1.11 | | 0.361 |
| Phosphatidylcholine (plasmalogen) | PCp 34:0 | 0.33 | 0.069 | 0.512 | 0.106 | 8.45 | | 1.71 | 2.03 | | 0.531 |
| Phosphatidylcholine (plasmalogen) | PCp 34:1 | 0.211 | 0.043 | 0.286 | 0.057 | 5.4 | | 1.11 | 1.14 | | 0.316 |
| Phosphatidylcholine (plasmalogen) | PCp 34:2 | 0.365 | 0.069 | 0.576 | 0.127 | 9.56 | | 2.81 | 2.29 | | 0.684 |
| Phosphatidylcholine (plasmalogen) | PCp 36:1 | 0.135 | 0.032 | 0.172 | 0.032 | 3.48 | | 0.839 | 0.689 | | 0.191 |
| Phosphatidylcholine (plasmalogen) | PCp 36:2 | 0.197 | 0.062 | 0.253 | 0.062 | 4.93 | | 0.999 | 1 | | 0.268 |
| Phosphatidylcholine (plasmalogen) | PCp 36:3 | 0.595 | 0.163 | 0.806 | 0.197 | 15 | | 3.25 | 3.18 | | 0.834 |
| Phosphatidylcholine (plasmalogen) | PCp 36:4 | 0.391 | 0.113 | 0.667 | 0.199 | 9.98 | | 2.86 | 2.62 | | 0.786 |
| Phosphatidylcholine (plasmalogen) | PCp 36:5 | 0.034 | 0.014 | 0.048 | 0.017 | 0.898 | | 0.397 | 0.189 | | 0.075 |
| Phosphatidylcholine (plasmalogen) | PCp 38:3 | 0.28 | 0.09 | 0.329 | 0.095 | 7.05 | | 1.98 | 1.32 | | 0.475 |
| Phosphatidylcholine (plasmalogen) | PCp 38:4 | 0.567 | 0.179 | 0.753 | 0.18 | 14.2 | | 3.17 | 2.96 | | 0.735 |
| Lyso phosphatidylethanolamine | LPE 16:0 | 15.4 | 2.74 | 16.1 | 2.91 | 1.05 | | 0.327 | 0.217 | | 0.07 |
| Lyso phosphatidylethanolamine | LPE 18:0 | 19.7 | 3.57 | 28.6 | 3.37 | 1.34 | | 0.432 | 0.38 | | 0.083 |
| Lyso phosphatidylethanolamine | LPE 18:1 | 14.7 | 5.9 | 16.1 | 4.46 | 0.998 | | 0.418 | 0.211 | | 0.061 |
| Lyso phosphatidylethanolamine | LPE 18:2 | 25.2 | 5.04 | 22.1 | 4.79 | 1.76 | | 0.771 | 0.296 | | 0.097 |
| Lyso phosphatidylethanolamine | LPE 20:4 | 9.89 | 2.35 | 7.33 | 1.69 | 0.646 | | 0.139 | 0.095 | | 0.019 |
| Lyso phosphatidylethanolamine | LPE 22:6 | 15.1 | 4.65 | 9.76 | 2.75 | 1 | | 0.318 | 0.13 | | 0.046 |
| Phosphatidylethanolamine | PE 32:1 | 0.114 | 0.1 | 0.151 | 0.08 | 0.082 | | 0.139 | 0.012 | | 0.022 |
| Phosphatidylethanolamine | PE 34:1 | 2.4 | 0.507 | 2.62 | 0.468 | 1.23 | | 1.03 | 0.17 | | 0.19 |
| Phosphatidylethanolamine | PE 34:2 | 5.05 | 1.21 | 5.34 | 1.14 | 2.75 | | 2.44 | 0.37 | | 0.442 |
| Phosphatidylethanolamine | PE 36:1 | 2.58 | 1 | 3.32 | 0.934 | 1.2 | | 0.619 | 0.184 | | 0.101 |
| Phosphatidylethanolamine | PE 36:2 | 17.9 | 4.52 | 20.4 | 4.42 | 8.9 | | 5.5 | 1.23 | | 0.923 |
| Phosphatidylethanolamine | PE 36:3 | 4.77 | 1.92 | 5.85 | 1.84 | 2.32 | | 1.62 | 0.344 | | 0.267 |
| Phosphatidylethanolamine | PE 36:4 | 4.65 | 0.861 | 4.57 | 0.883 | 2.34 | | 1.85 | 0.318 | | 0.401 |
| Phosphatidylethanolamine | PE 36:5 | 0.699 | 0.325 | 0.591 | 0.234 | 0.364 | | 0.282 | 0.04 | | 0.042 |
| Phosphatidylethanolamine | PE 38:3 | 1.57 | 0.627 | 1.88 | 0.544 | 0.813 | | 0.6 | 0.122 | | 0.122 |
| Phosphatidylethanolamine | PE 38:4 | 18.2 | 3.95 | 16.8 | 3.26 | 8.82 | | 5.28 | 1.06 | | 0.984 |
| Phosphatidylethanolamine | PE 38:5 | 5.62 | 1.14 | 5.17 | 0.86 | 2.79 | | 1.74 | 0.333 | | 0.329 |
| Phosphatidylethanolamine | PE 38:6 | 17.4 | 5.18 | 14.5 | 4.8 | 9.81 | | 9.71 | 1.15 | | 1.76 |
| Phosphatidylethanolamine | PE 40:4 | 0.328 | 0.145 | 0.321 | 0.116 | 0.167 | | 0.135 | 0.02 | | 0.02 |
| Phosphatidylethanolamine | PE 40:5 | 1.48 | 0.371 | 1.34 | 0.306 | 0.751 | | 0.507 | 0.09 | | 0.099 |
| Phosphatidylethanolamine | PE 40:6 | 9.73 | 2.33 | 8.43 | 2.14 | 5.23 | | 4.24 | 0.61 | | 0.743 |
| Phosphatidylethanolamine | PE 40:7 | 1.66 | 0.43 | 1.72 | 0.377 | 0.875 | | 0.773 | 0.122 | | 0.159 |
| Phosphatidylethanolamine (plasmalogen) | PEp 34:1 | 0.105 | 0.107 | 0.189 | 0.148 | 0.034 | | 0.025 | 0.008 | | 0.004 |
| Phosphatidylethanolamine (plasmalogen) | PEp 34:2 | 0.106 | 0.067 | 0.232 | 0.127 | 0.045 | | 0.028 | 0.011 | | 0.005 |
| Phosphatidylethanolamine (plasmalogen) | PEp 36:2 | 0.327 | 0.194 | 0.356 | 0.142 | 0.128 | | 0.059 | 0.018 | | 0.008 |
| Phosphatidylethanolamine (plasmalogen) | PEp 36:3 | 0.921 | 0.47 | 1.18 | 0.547 | 0.386 | | 0.162 | 0.058 | | 0.021 |
| Phosphatidylethanolamine (plasmalogen) | PEp 36:4 | 0.41 | 0.227 | 0.59 | 0.27 | 0.164 | | 0.054 | 0.028 | | 0.008 |
| Phosphatidylethanolamine (plasmalogen) | PEp 38:4 | 0.683 | 0.393 | 0.869 | 0.511 | 0.273 | | 0.121 | 0.04 | | 0.012 |
| Phosphatidylethanolamine (plasmalogen) | PEp 38:5 | 2.31 | 0.907 | 2.62 | 1.04 | 0.948 | | 0.29 | 0.127 | | 0.033 |
| Phosphatidylethanolamine (plasmalogen) | PEp 38:6 | 0.532 | 0.282 | 0.585 | 0.241 | 0.219 | | 0.077 | 0.03 | | 0.011 |
| Phosphatidylethanolamine (plasmalogen) | PEp 40:6 | 0.411 | 0.249 | 0.499 | 0.208 | 0.164 | | 0.068 | 0.024 | | 0.008 |
| Phosphatidylinositol | PI 32:1 | 3.37 | 1.78 | 3.51 | 1.68 | 1.78 | | 1.29 | 0.357 | | 0.21 |
| Phosphatidylinositol | PI 34:1 | 6.01 | 1.09 | 7.78 | 1.65 | 3.19 | | 1.79 | 0.772 | | 0.229 |
| Phosphatidylinositol | PI 34:2 | 4.66 | 1.43 | 4.4 | 0.858 | 2.58 | | 1.92 | 0.446 | | 0.158 |
| Phosphatidylinositol | PI 36:1 | 8.42 | 2.43 | 8.71 | 2.18 | 4.45 | | 2.73 | 0.866 | | 0.288 |
| Phosphatidylinositol | PI 36:2 | 18.7 | 5.27 | 19 | 4.78 | 9.94 | | 5.88 | 1.88 | | 0.58 |
| Phosphatidylinositol | PI 36:3 | 2.6 | 1.01 | 2.54 | 0.677 | 1.39 | | 0.955 | 0.253 | | 0.08 |
| Phosphatidylinositol | PI 36:4 | 4.11 | 1.02 | 3.61 | 0.774 | 2.2 | | 1.15 | 0.372 | | 0.152 |
| Phosphatidylinositol | PI 38:3 | 8.4 | 2.61 | 7.94 | 2.27 | 4.32 | | 2 | 0.823 | | 0.396 |
| Phosphatidylinositol | PI 38:4 | 35.6 | 7.08 | 35.1 | 7.15 | 18.2 | | 8.5 | 3.51 | | 1.14 |
| Phosphatidylinositol | PI 38:5 | 1.55 | 0.405 | 1.47 | 0.32 | 0.826 | | 0.453 | 0.15 | | 0.058 |
| Phosphatidylinositol | PI 38:6 | 3.29 | 1.11 | 3 | 1.11 | 1.73 | | 1.06 | 0.302 | | 0.131 |
| Phosphatidylinositol | PI 40:5 | 1.15 | 0.263 | 0.962 | 0.342 | 0.601 | | 0.305 | 0.097 | | 0.044 |
| Phosphatidylinositol | PI 40:6 | 2.11 | 0.53 | 2.01 | 0.73 | 1.12 | | 0.604 | 0.199 | | 0.075 |
| Phosphatidylserine | PS 36:1 | 47.7 | 8.95 | 91.7 | 4.78 | 0.427 | | 0.355 | 0.26 | | 0.075 |
| Phosphatidylserine | PS 38:4 | 52.3 | 8.95 | 8.29 | 4.78 | 0.435 | | 0.298 | 0.024 | | 0.018 |
| Sphingomyelin | SM 32:1 | 2.05 | 0.344 | 2.32 | 0.378 | 14.5 | | 5.14 | 3.17 | | 0.935 |
| Sphingomyelin | SM 33:1 | 2.22 | 0.433 | 2.47 | 0.4 | 15.8 | | 5.81 | 3.41 | | 1.07 |
| Sphingomyelin | SM 34:1 | 26.8 | 2.03 | 31 | 2.55 | 185 | | 39.4 | 42 | | 9.79 |
| Sphingomyelin | SM 34:2 | 3.88 | 0.404 | 4.36 | 0.409 | 27 | | 7.41 | 5.9 | | 1.45 |
| Sphingomyelin | SM 36:1 | 4.6 | 0.77 | 4.3 | 0.71 | 32 | | 8.75 | 5.9 | | 1.88 |
| Sphingomyelin | SM 36:2 | 2.01 | 0.346 | 1.88 | 0.301 | 14 | | 4.04 | 2.57 | | 0.805 |
| Sphingomyelin | SM 37:1 | 0.842 | 0.081 | 0.993 | 0.097 | 5.85 | | 1.47 | 1.35 | | 0.334 |
| Sphingomyelin | SM 38:1 | 4.26 | 0.546 | 3.65 | 0.572 | 29.6 | | 7.82 | 4.98 | | 1.49 |
| Sphingomyelin | SM 38:2 | 1.4 | 0.156 | 1.4 | 0.176 | 9.75 | | 2.51 | 1.92 | | 0.579 |
| Sphingomyelin | SM 39:1 | 1.75 | 0.306 | 1.5 | 0.292 | 12.3 | | 3.9 | 2.06 | | 0.681 |
| Sphingomyelin | SM 40:1 | 8.24 | 0.855 | 7.06 | 0.831 | 56.7 | | 12.4 | 9.57 | | 2.49 |
| Sphingomyelin | SM 40:2 | 6.63 | 0.516 | 6.1 | 0.587 | 46.1 | | 11.8 | 8.28 | | 2.08 |
| Sphingomyelin | SM 41:1 | 3.32 | 0.398 | 2.72 | 0.415 | 23.2 | | 6.5 | 3.76 | | 1.21 |
| Sphingomyelin | SM 41:2 | 3.16 | 0.443 | 2.82 | 0.441 | 22.3 | | 7.16 | 3.89 | | 1.21 |
| Sphingomyelin | SM 42:1 | 4.4 | 0.553 | 3.83 | 0.481 | 30.2 | | 6.69 | 5.19 | | 1.31 |
| Sphingomyelin | SM 42:2 | 16.2 | 1.43 | 15.6 | 1.24 | 112 | | 26.4 | 21 | | 4.71 |
| Sphingomyelin | SM 42:3 | 7.13 | 0.871 | 7.09 | 0.829 | 49.7 | | 14.1 | 9.57 | | 2.27 |
| Sphingomyelin | SM 43:1 | 0.196 | 0.035 | 0.177 | 0.036 | 1.36 | | 0.38 | 0.245 | | 0.079 |
| Sphingomyelin | SM 43:2 | 0.763 | 0.228 | 0.677 | 0.191 | 5.31 | | 1.87 | 0.925 | | 0.32 |
| Sphingomyelin | SM 44:2 | 0.086 | 0.012 | 0.109 | 0.021 | 0.594 | | 0.158 | 0.145 | | 0.038 |
| Diglyceride | DG 34:0 | 6.04 | 1.46 | 14 | 1.83 | 4.08 | | 1.39 | 2.04 | | 0.221 |
| Diglyceride | DG 34:1 | 8.5 | 2.92 | 5.24 | 2.4 | 5.99 | | 2.88 | 0.794 | | 0.44 |
| Diglyceride | DG 34:2 | 48.3 | 8.22 | 55.9 | 5.35 | 31.8 | | 2.56 | 8.14 | | 0.502 |
| Diglyceride | DG 36:0 | 2.33 | 0.636 | 4.79 | 0.979 | 1.58 | | 0.588 | 0.7 | | 0.149 |
| Diglyceride | DG 36:1 | 1.74 | 0.705 | 2.22 | 0.442 | 1.21 | | 0.639 | 0.333 | | 0.105 |
| Diglyceride | DG 36:2 | 18.8 | 4.64 | 10.3 | 2.94 | 13.3 | | 5.81 | 1.55 | | 0.618 |
| Diglyceride | DG 36:3 | 10.7 | 2.34 | 5.34 | 1.71 | 7.42 | | 2.72 | 0.804 | | 0.322 |
| Diglyceride | DG 36:4 | 3.58 | 0.959 | 2.26 | 0.63 | 2.48 | | 1.04 | 0.339 | | 0.126 |
| Triglyceride | TG 44:0 | 0.065 | 0.082 | 0.109 | 0.05 | 0.59 | | 0.958 | 0.087 | | 0.046 |
| Triglyceride | TG 44:1 | 0.076 | 0.136 | 0.083 | 0.094 | 0.723 | | 1.53 | 0.069 | | 0.074 |
| Triglyceride | TG 45:0 | 0.039 | 0.014 | 0.083 | 0.032 | 0.3 | | 0.174 | 0.062 | | 0.016 |
| Triglyceride | TG 46:0 | 0.126 | 0.134 | 0.176 | 0.132 | 1.17 | | 1.57 | 0.158 | | 0.174 |
| Triglyceride | TG 46:1 | 0.311 | 0.333 | 0.313 | 0.237 | 2.9 | | 3.87 | 0.295 | | 0.327 |
| Triglyceride | TG 46:2 | 0.154 | 0.175 | 0.148 | 0.109 | 1.4 | | 2 | 0.13 | | 0.117 |
| Triglyceride | TG 47:0 | 0.051 | 0.028 | 0.111 | 0.043 | 0.42 | | 0.328 | 0.087 | | 0.035 |
| Triglyceride | TG 47:1 | 0.078 | 0.059 | 0.102 | 0.05 | 0.691 | | 0.689 | 0.089 | | 0.067 |
| Triglyceride | TG 48:0 | 0.276 | 0.157 | 0.458 | 0.255 | 2.38 | | 1.85 | 0.427 | | 0.451 |
| Triglyceride | TG 48:1 | 1.55 | 1.08 | 1.56 | 0.965 | 14.1 | | 13.5 | 1.6 | | 1.93 |
| Triglyceride | TG 48:2 | 1.02 | 0.661 | 0.926 | 0.48 | 9.2 | | 8.23 | 0.917 | | 0.967 |
| Triglyceride | TG 48:3 | 0.297 | 0.196 | 0.27 | 0.134 | 2.57 | | 2.37 | 0.243 | | 0.194 |
| Triglyceride | TG 48:4 | 0.06 | 0.044 | 0.064 | 0.042 | 0.501 | | 0.505 | 0.052 | | 0.036 |
| Triglyceride | TG 50:0 | 0.167 | 0.076 | 0.311 | 0.14 | 1.41 | | 0.843 | 0.278 | | 0.23 |
| Triglyceride | TG 50:1 | 4.14 | 1.55 | 4.53 | 1.61 | 35.3 | | 22.8 | 4.56 | | 4.67 |
| Triglyceride | TG 50:2 | 5.52 | 1.78 | 5.23 | 1.57 | 47.2 | | 28.8 | 5.22 | | 4.93 |
| Triglyceride | TG 50:3 | 2 | 0.571 | 1.69 | 0.411 | 16.9 | | 9.75 | 1.66 | | 1.45 |
| Triglyceride | TG 50:4 | 0.48 | 0.179 | 0.427 | 0.136 | 4.02 | | 2.49 | 0.392 | | 0.287 |
| Triglyceride | TG 50:5 | 0.094 | 0.058 | 0.087 | 0.039 | 0.768 | | 0.642 | 0.074 | | 0.043 |
| Triglyceride | TG 51:1 | 0.277 | 0.139 | 0.302 | 0.125 | 2.46 | | 1.82 | 0.294 | | 0.272 |
| Triglyceride | TG 51:2 | 0.755 | 0.267 | 0.693 | 0.219 | 6.41 | | 3.72 | 0.676 | | 0.582 |
| Triglyceride | TG 51:3 | 0.409 | 0.123 | 0.369 | 0.108 | 3.42 | | 1.82 | 0.347 | | 0.258 |
| Triglyceride | TG 52:0 | 0.038 | 0.012 | 0.098 | 0.035 | 0.294 | | 0.115 | 0.076 | | 0.024 |
| Triglyceride | TG 52:1 | 1.36 | 0.532 | 1.57 | 0.518 | 11.9 | | 7.98 | 1.47 | | 1.19 |
| Triglyceride | TG 52:2 | 23.4 | 3.34 | 24.8 | 3.22 | 186 | | 73 | 22.8 | | 15.2 |
| Triglyceride | TG 52:3 | 18.4 | 1.67 | 17.3 | 1.43 | 145 | | 52.6 | 15.8 | | 10.4 |
| Triglyceride | TG 52:4 | 6.23 | 0.958 | 5.52 | 0.862 | 49.1 | | 18.2 | 5 | | 3.27 |
| Triglyceride | TG 52:5 | 1.39 | 0.4 | 1.78 | 0.406 | 10.9 | | 4.65 | 1.48 | | 0.652 |
| Triglyceride | TG 52:6 | 0.34 | 0.131 | 0.371 | 0.113 | 2.72 | | 1.47 | 0.314 | | 0.158 |
| Triglyceride | TG 52:7 | 0.095 | 0.033 | 0.126 | 0.036 | 0.744 | | 0.342 | 0.103 | | 0.045 |
| Triglyceride | TG 53:2 | 0.508 | 0.144 | 0.507 | 0.135 | 4.23 | | 2.18 | 0.47 | | 0.325 |
| Triglyceride | TG 53:3 | 0.504 | 0.113 | 0.468 | 0.102 | 4.08 | | 1.82 | 0.423 | | 0.251 |
| Triglyceride | TG 53:4 | 0.212 | 0.041 | 0.208 | 0.039 | 1.69 | | 0.689 | 0.181 | | 0.094 |
| Triglyceride | TG 54:0 | 0.016 | 0.012 | 0.024 | 0.016 | 0.132 | | 0.141 | 0.018 | | 0.01 |
| Triglyceride | TG 54:1 | 0.121 | 0.056 | 0.137 | 0.04 | 1.01 | | 0.641 | 0.119 | | 0.064 |
| Triglyceride | TG 54:2 | 1.9 | 0.401 | 2.02 | 0.32 | 15.1 | | 6.15 | 1.78 | | 0.977 |
| Triglyceride | TG 54:3 | 8.03 | 2.57 | 8.66 | 2.2 | 59.8 | | 20.1 | 7.19 | | 3.22 |
| Triglyceride | TG 54:4 | 6.43 | 2.66 | 5.95 | 2.19 | 47.2 | | 16.2 | 4.92 | | 2.36 |
| Triglyceride | TG 54:5 | 3.61 | 1.5 | 3.23 | 1.33 | 26.7 | | 8.51 | 2.68 | | 1.32 |
| Triglyceride | TG 54:6 | 1.56 | 0.654 | 1.37 | 0.623 | 11.7 | | 4.01 | 1.13 | | 0.554 |
| Triglyceride | TG 54:7 | 0.684 | 0.26 | 0.752 | 0.214 | 5.09 | | 1.68 | 0.618 | | 0.268 |
| Triglyceride | TG 56:2 | 0.116 | 0.114 | 0.105 | 0.032 | 0.858 | | 0.651 | 0.085 | | 0.035 |
| Triglyceride | TG 56:3 | 0.22 | 0.129 | 0.217 | 0.076 | 1.63 | | 0.787 | 0.176 | | 0.074 |
| Triglyceride | TG 56:4 | 0.48 | 0.152 | 0.498 | 0.142 | 3.59 | | 1.13 | 0.408 | | 0.169 |
| Triglyceride | TG 56:5 | 0.918 | 0.287 | 0.962 | 0.251 | 6.78 | | 2.01 | 0.797 | | 0.34 |
| Triglyceride | TG 56:6 | 1.67 | 0.37 | 1.59 | 0.34 | 12.5 | | 3.42 | 1.32 | | 0.588 |
| Triglyceride | TG 56:7 | 1.74 | 0.537 | 1.52 | 0.442 | 13 | | 4.32 | 1.27 | | 0.667 |
| Triglyceride | TG 56:8 | 0.753 | 0.267 | 0.777 | 0.267 | 5.46 | | 1.49 | 0.622 | | 0.263 |
| Triglyceride | TG 56:9 | 0.191 | 0.109 | 0.243 | 0.101 | 1.34 | | 0.476 | 0.188 | | 0.06 |
| Triglyceride | TG 58:6 | 0.176 | 0.041 | 0.207 | 0.046 | 1.34 | | 0.461 | 0.176 | | 0.101 |
| Triglyceride | TG 58:7 | 0.277 | 0.074 | 0.304 | 0.082 | 2.07 | | 0.661 | 0.249 | | 0.113 |
| Triglyceride | TG 58:8 | 0.433 | 0.189 | 0.43 | 0.182 | 3.1 | | 1.11 | 0.343 | | 0.167 |
| Triglyceride | TG 58:9 | 0.259 | 0.13 | 0.236 | 0.117 | 1.81 | | 0.632 | 0.182 | | 0.082 |
| Cholesteryl ester | CE 14:0 | 0.297 | 0.091 | 0.324 | 0.087 | 14.6 | | 6.36 | 2.8 | | 1.03 |
| Cholesteryl ester | CE 15:0 | 0.132 | 0.044 | 0.142 | 0.039 | 6.54 | | 2.92 | 1.23 | | 0.462 |
| Cholesteryl ester | CE 16:0 | 6.01 | 1.41 | 5.67 | 0.306 | 288 | | 95.7 | 48 | | 10.6 |
| Cholesteryl ester | CE 16:1 | 2.3 | 0.824 | 2.32 | 0.791 | 113 | | 52.3 | 19.9 | | 8.02 |
| Cholesteryl ester | CE 16:2 | 0.038 | 0.012 | 0.038 | 0.009 | 1.89 | | 0.937 | 0.324 | | 0.104 |
| Cholesteryl ester | CE 17:0 | 0.166 | 0.064 | 0.156 | 0.041 | 8.28 | | 4.16 | 1.35 | | 0.485 |
| Cholesteryl ester | CE 17:1 | 0.224 | 0.068 | 0.222 | 0.06 | 11 | | 4.73 | 1.9 | | 0.649 |
| Cholesteryl ester | CE 18:0 | 0.664 | 0.265 | 0.631 | 0.114 | 32.7 | | 16.6 | 5.39 | | 1.69 |
| Cholesteryl ester | CE 18:1 | 20.1 | 1.12 | 19.9 | 0.996 | 954 | | 213 | 168 | | 38.1 |
| Cholesteryl ester | CE 18:2 | 50.1 | 3.64 | 51.1 | 3.44 | 2358 | | 419 | 428 | | 79.1 |
| Cholesteryl ester | CE 18:3 | 3.15 | 0.675 | 2.9 | 0.516 | 154 | | 60.7 | 24.6 | | 7.34 |
| Cholesteryl ester | CE 18:4 | 0.06 | 0.037 | 0.057 | 0.026 | 3.08 | | 2.62 | 0.48 | | 0.258 |
| Cholesteryl ester | CE 20:1 | 0.03 | 0.012 | 0.03 | 0.012 | 1.52 | | 0.837 | 0.25 | | 0.103 |
| Cholesteryl ester | CE 20:2 | 0.043 | 0.004 | 0.04 | 0.005 | 2.04 | | 0.508 | 0.342 | | 0.1 |
| Cholesteryl ester | CE 20:3 | 1.15 | 0.325 | 1.16 | 0.333 | 55.3 | | 19.4 | 10 | | 4.14 |
| Cholesteryl ester | CE 20:4 | 11.1 | 1.94 | 11 | 2.11 | 529 | | 152 | 93.5 | | 27 |
| Cholesteryl ester | CE 20:5 | 2.64 | 0.977 | 2.54 | 0.88 | 132 | | 71.8 | 21.6 | | 8.75 |
| Cholesteryl ester | CE 22:5 | 0.096 | 0.015 | 0.094 | 0.017 | 4.6 | | 1.38 | 0.803 | | 0.245 |
| Cholesteryl ester | CE 22:6 | 1.76 | 0.45 | 1.67 | 0.406 | 86.2 | | 34.3 | 14.4 | | 5.03 |
