# Supplementary material for: Lipid profiling of suction blister fluid: comparison of lipids in interstitial fluid and plasma
Source: Lipids Health Dis. 2019 Aug 24;18:164. doi: 10.1186/s12944-019-1107-3 (PMC6708155; doi:10.1186/s12944-019-1107-3)
Supplement: Supplementary file 6 — Table S3. Predictive variable importance for the OPLS-DA projection (VIP score) and group comparison of lipid species in plasma and SBF using the Mann–Whitney U test. (DOCX 29 kb) [file 12944_2019_1107_MOESM6_ESM.docx]

**Additional file 6: Table S3: Predictive variable importance for the OPLS-DA projection (VIP score) and group comparison of lipid species in plasma and SBF using the Mann–Whitney *U* test.**

| Lipid species | VIP score | *P* value |
| --- | --- | --- |
| DG 34:0 | 2.733 | 0.0000003 |
| LPCp 16:0 | 2.627 | 0.0000003 |
| DG 36:0 | 2.497 | 0.0000003 |
| DG 36:3 | 2.352 | 0.0000033 |
| LPE 18:0 | 2.340 | 0.0000028 |
| TG 52:0 | 2.316 | 0.0000009 |
| PC 32:0 | 2.240 | 0.0000009 |
| PCp 34:2 | 2.169 | 0.0000094 |
| DG 36:2 | 2.164 | 0.0000045 |
| PCp 34:0 | 2.117 | 0.0000224 |
| SM 37:1 | 2.035 | 0.0000671 |
| SM 34:1 | 2.016 | 0.0000671 |
| TG 45:0 | 2.006 | 0.0000391 |
| PCp 36:4 | 1.999 | 0.0000874 |
| LPC 22:5 | 1.946 | 0.0000513 |
| TG 47:0 | 1.903 | 0.0000258 |
| DG 36:4 | 1.853 | 0.0000448 |
| PCp 34:1 | 1.794 | 0.0001889 |
| SM 40:1 | 1.744 | 0.0001889 |
| SM 41:1 | 1.736 | 0.0003100 |
| PI 34:1 | 1.668 | 0.0023870 |
| SM 44:2 | 1.664 | 0.0007109 |
| TG 50:0 | 1.664 | 0.0007109 |
| LPE 22:6 | 1.629 | 0.0007109 |
| PEp 34:2 | 1.587 | 0.0017349 |
| LPC 22:6 | 1.584 | 0.0003100 |
| PCp 36:3 | 1.564 | 0.0044068 |
| DG 34:1 | 1.563 | 0.0008936 |
| LPE 20:4 | 1.522 | 0.0013959 |
| SM 34:2 | 1.519 | 0.0012504 |
| SM 38:1 | 1.467 | 0.0032569 |
| PCp 36:1 | 1.464 | 0.0017349 |
| SM 42:1 | 1.461 | 0.0032569 |
| PC 34:4 | 1.421 | 0.0053660 |
| PCp 38:4 | 1.407 | 0.0059132 |
| DG 34:2 | 1.398 | 0.0044068 |
| PC 34:3 | 1.391 | 0.0078689 |
| SM 40:2 | 1.320 | 0.0078689 |
| TG 52:5 | 1.279 | 0.0006332 |
| LPC 20:3 | 1.259 | 0.0209093 |
| PCp 36:5 | 1.243 | 0.0161931 |
| PC 36:5 | 1.229 | 0.0192190 |
| PCp 36:2 | 1.207 | 0.0044068 |
| TG 52:7 | 1.185 | 0.0148436 |
| TG 48:0 | 1.172 | 0.0078689 |
| TG 52:4 | 1.162 | 0.0048651 |
| SM 39:1 | 1.155 | 0.0086394 |
| PC 38:6 | 1.138 | 0.0044068 |
| PC 32:2 | 1.132 | 0.0428807 |
| TG 58:6 | 1.107 | 0.0498099 |
| PC 34:0 | 1.099 | 0.0113709 |
| SM 41:2 | 1.049 | 0.0176493 |
| DG 36:1 | 1.045 | 0.0340241 |
| PEp 36:4 | 1.044 | 0.0290311 |
| PE 36:1 | 1.016 | 0.0044068 |
| SM 32:1 | 0.988 | 0.0397335 |
| LPE 18:2 | 0.984 | 0.0428807 |
| TG 52:3 | 0.960 | 0.0462361 |
| PC 36:3 | 0.945 | 0.0713254 |
| LPC 16:0 | 0.940 | 0.0935726 |
| TG 50:3 | 0.927 | 0.0935726 |
| SM 33:1 | 0.870 | 0.0340241 |
| PEp 34:1 | 0.868 | 0.0290311 |
| LPC 18:2 | 0.863 | 0.0664999 |
| TG 44:0 | 0.852 | 0.0000766 |
| PI 40:5 | 0.836 | 0.1210716 |
| PI 36:4 | 0.829 | 0.1638926 |
| PCp 38:3 | 0.820 | 0.1136656 |
| PE 38:6 | 0.807 | 0.0576545 |
| CE 20:2 | 0.802 | 0.0619467 |
| PEp 36:3 | 0.789 | 0.1545226 |
| PE 40:6 | 0.786 | 0.0428807 |
| SM 42:2 | 0.772 | 0.0818374 |
| PE 38:3 | 0.770 | 0.0498099 |
| PE 36:3 | 0.761 | 0.0397335 |
| PC 38:4 | 0.754 | 0.0576545 |
| LPC 14:0 | 0.751 | 0.1066210 |
| TG 54:0 | 0.741 | 0.0764341 |
| TG 52:2 | 0.728 | 0.0619467 |
| TG 56:9 | 0.723 | 0.0428807 |
| SM 43:1 | 0.717 | 0.0999271 |
| PE 38:5 | 0.712 | 0.1288499 |
| PE 36:2 | 0.704 | 0.0818374 |
| LPC 16:1 | 0.690 | 0.2056764 |
| LPC 20:5 | 0.682 | 0.1136656 |
| CE 18:3 | 0.662 | 0.2417483 |
| LPC 20:4 | 0.659 | 0.0818374 |
| PC 30:0 | 0.651 | 0.1066210 |
| PE 36:5 | 0.640 | 0.2172382 |
| PE 34:1 | 0.640 | 0.2417483 |
| PEp 38:4 | 0.637 | 0.2681422 |
| PE 32:1 | 0.625 | 0.2056764 |
| PE 40:5 | 0.621 | 0.2417483 |
| TG 58:7 | 0.614 | 0.4107335 |
| PC 40:7 | 0.605 | 0.1638926 |
| TG 56:7 | 0.599 | 0.1839070 |
| TG 50:4 | 0.599 | 0.2681422 |
| TG 47:1 | 0.594 | 0.0246822 |
| PEp 40:6 | 0.585 | 0.1839070 |
| SM 36:2 | 0.584 | 0.0536125 |
| TG 52:1 | 0.580 | 0.1839070 |
| SM 36:1 | 0.578 | 0.1066210 |
| SM 43:2 | 0.567 | 0.1736845 |
| PE 38:4 | 0.546 | 0.1288499 |
| TG 54:2 | 0.536 | 0.2172382 |
| PEp 38:5 | 0.536 | 0.2964512 |
| TG 51:3 | 0.533 | 0.1945684 |
| LPC 18:1 | 0.513 | 0.3425403 |
| TG 54:6 | 0.511 | 0.0290311 |
| TG 46:0 | 0.490 | 0.0340241 |
| PC 34:1 | 0.484 | 0.3588705 |
| TG 53:3 | 0.476 | 0.2056764 |
| CE 14:0 | 0.463 | 0.3588705 |
| TG 54:5 | 0.452 | 0.1638926 |
| PI 38:5 | 0.450 | 0.4107335 |
| PC 40:4 | 0.431 | 0.3266931 |
| TG 54:1 | 0.430 | 0.1455653 |
| PI 34:2 | 0.428 | 0.6808513 |
| LPE 16:0 | 0.407 | 0.7278217 |
| LPC 17:0 | 0.406 | 0.4476573 |
| TG 54:3 | 0.401 | 0.2292602 |
| TG 54:7 | 0.398 | 0.1370111 |
| CE 15:0 | 0.379 | 0.4107335 |
| TG 51:2 | 0.378 | 0.2547075 |
| PC 36:1 | 0.374 | 0.3425403 |
| PI 38:6 | 0.369 | 0.6808513 |
| CE 18:2 | 0.356 | 0.4476573 |
| LPC 18:0 | 0.356 | 0.7517095 |
| TG 50:1 | 0.355 | 0.4863980 |
| TG 48:3 | 0.350 | 0.7041954 |
| CE 16:0 | 0.349 | 0.4863980 |
| TG 54:4 | 0.341 | 0.6578088 |
| LPE 18:1 | 0.341 | 0.1136656 |
| PEp 38:6 | 0.340 | 0.3588705 |
| TG 50:5 | 0.332 | 0.8247263 |
| PE 40:7 | 0.327 | 0.6350871 |
| TG 48:2 | 0.327 | 0.7041954 |
| PC 38:2 | 0.315 | 0.5690204 |
| TG 56:5 | 0.311 | 0.3425403 |
| PI 38:3 | 0.308 | 0.5477511 |
| PE 34:2 | 0.307 | 0.4668047 |
| LPC 18:3 | 0.304 | 0.2820559 |
| TG 52:6 | 0.301 | 0.3113300 |
| CE 22:6 | 0.299 | 0.3756818 |
| PC 38:3 | 0.276 | 0.6350871 |
| TG 56:6 | 0.272 | 0.5906763 |
| CE 18:1 | 0.266 | 0.8247263 |
| PC 40:6 | 0.265 | 0.4863980 |
| PEp 36:2 | 0.259 | 0.5064276 |
| TG 50:2 | 0.257 | 0.4668047 |
| TG 56:2 | 0.246 | 0.1638926 |
| TG 51:1 | 0.245 | 0.5477511 |
| TG 56:4 | 0.232 | 0.6808513 |
| TG 58:9 | 0.222 | 0.6578088 |
| CE 18:4 | 0.206 | 0.8001841 |
| CE 20:5 | 0.198 | 0.8001841 |
| CE 17:0 | 0.196 | 0.9747603 |
| PI 36:3 | 0.195 | 0.5690204 |
| CE 22:5 | 0.193 | 0.8992931 |
| PC 36:4 | 0.185 | 0.2417483 |
| PC 38:5 | 0.183 | 0.7041954 |
| TG 56:8 | 0.169 | 0.8001841 |
| TG 46:2 | 0.163 | 0.5268825 |
| PI 40:6 | 0.156 | 0.2964512 |
| PI 36:1 | 0.149 | 0.6578088 |
| CE 18:0 | 0.149 | 0.6127039 |
| PC 40:5 | 0.136 | 0.6578088 |
| PI 32:1 | 0.125 | 0.6350871 |
| CE 16:1 | 0.119 | 0.9495458 |
| TG 58:8 | 0.093 | 0.9495458 |
| PE 40:4 | 0.089 | 0.9747603 |
| TG 46:1 | 0.072 | 0.3929708 |
| SM 42:3 | 0.071 | 1.0000000 |
| TG 48:4 | 0.070 | 0.7517095 |
| PI 38:4 | 0.070 | 0.7278217 |
| PC 34:2 | 0.063 | 0.7278217 |
| PI 36:2 | 0.062 | 0.5906763 |
| PC 36:2 | 0.061 | 0.7517095 |
| TG 53:4 | 0.058 | 0.8247263 |
| PE 36:4 | 0.056 | 0.8001841 |
| TG 56:3 | 0.051 | 0.3588705 |
| TG 53:2 | 0.050 | 0.9495458 |
| TG 48:1 | 0.048 | 0.8992931 |
| CE 17:1 | 0.026 | 0.8992931 |
| CE 20:1 | 0.012 | 0.8001841 |
| CE 16:2 | 0.010 | 0.7758377 |
| TG 44:1 | 0.010 | 0.0999271 |
| PC 32:1 | 0.006 | 0.8992931 |
| CE 20:4 | 0.005 | 0.8494411 |
| CE 20:3 | 0.004 | 0.9243817 |
| SM 38:2 | 0.004 | 0.8494411 |
